# Upregulation of FAM84B during prostate cancer progression

**DOI:** 10.18632/oncotarget.15168

**Published:** 2017-02-07

**Authors:** Nicholas Wong, Yan Gu, Anil Kapoor, Xiaozeng Lin, Diane Ojo, Fengxiang Wei, Judy Yan, Jason de Melo, Pierre Major, Geoffrey Wood, Tariq Aziz, Jean-Claude Cutz, Michael Bonert, Arthur J. Patterson, Damu Tang

**Affiliations:** ^1^ Division of Nephrology, Department of Medicine, McMaster University, Hamilton, Ontario, Canada; ^2^ Father Sean O’Sullivan Research Institute, Hamilton, Ontario, Canada; ^3^ The Hamilton Center for Kidney Research, St. Joseph's Hospital, Hamilton, Ontario, Canada; ^4^ Department of Surgery, McMaster University, Hamilton, Ontario, Canada; ^5^ The Genetics Laboratory, Longgang District Maternity and Child Healthcare Hospital, Longgang District, Shenzhen, Guangdong, P.R. China; ^6^ Division of Medical Oncology, Department of Oncology, McMaster University, Ontario, Canada; ^7^ Department of Veterinary Pathology, University of Guelph, Guelph, Ontario, Canada; ^8^ Department of Pathology and Molecular Medicine, McMaster University, Hamilton, Ontario, Canada

**Keywords:** FAM84B, prostate cancer, prostate cancer stem cells, metastasis, castration resistant prostate cancer

## Abstract

Although the *FAM84B* gene lies within chromosome 8q24, a locus frequently altered in prostate cancer (PC), its alteration during prostate tumorigenesis has not been well studied. We report here FAM84B upregulation in DU145 cell-derived prostate cancer stem-like cells (PCSLCs) and DU145 cell-produced lung metastases compared to subcutaneous xenograft tumors. FAM84B protein was detected in bone metastases and primary PCs. Nanostring examination of 7 pairs of tumor adjacent normal and PC tissues revealed elevations in FAM84B mRNA levels in all carcinomas. Furthermore, through analysis of FAM84B expression using large datasets within the Gene Expression Omnibus and Oncomine^TM^ database, we demonstrate significant increases in FAM84B mRNA in 343 primary PCs versus 181 normal tissues, and elevations in the FAM84B gene copy number (GCN) in 171 primary PCs versus 61 normal tissues. While FAM84B was not detected at higher levels via immunohistochemistry in high grade (Gleason score/GS 8-10) tumors compared to GS6-7 PCs, analyses of FAM84B mRNA and GCN using datasets within the cBioPortal database demonstrated FAM84B upregulation in 12% (67/549) of primary PCs and 18% (73/412) of metastatic castration resistant PCs (mCRPCs), and GCN increases in 4.8% (26/546) of primary PCs and 26% (121/467) of mCRPCs, revealing an association of the aforementioned changes with CRPC development. Of note, an increase in FAM84B expression was observed in xenograft CRPCs produced by LNCaP cells. Furthermore, FAM84B upregulation and GCN increases correlate with decreases in disease free survival and overall survival. Collectively, we demonstrate a novel association of FAM84B with PC tumorigenesis and CRPC progression.

## INTRODUCTION

Prostate cancer (PC) is the most frequently diagnosed cancer type in men in the developed world [1]. The disease likely originates from high grade prostatic intra-epithelial neoplasia (HGPIN) and progresses to metastatic PC with bone as the most common site [2]. Organ-confined tumors can be managed through multiple means, such as active surveillance, surgery, or radiation. Patients with advanced and metastatic disease can be treated with androgen deprivation therapy (ADT), a strategy pioneered by Charles Huggins in the 1940s [3, 4] and remains the current standard of care. The treatment however is not curative, as resistant tumors known as castration resistant PCs (CRPCs) inevitably develop. While CRPC development is likely mediated by multiple mechanisms, the best understood property of this progression is its dependency of androgen receptor (AR) signalling. Despite low serum testosterone levels following castration (< 50 ng/ml), persistent AR signalling continues, making it a critical contributing factor in CRPC development [5–7]. However, it appears that AR-independent mechanisms are also involved in CRPC acquisition, like in the case of AR-negative neuroendocrine PCs (NEPCs) [8], an aggressive type of CRPC. ADT [9, 10] and inhibition of AR signalling through both abiraterone and enzalutamide [11, 12] has been shown to induce NEPCs. Nevertheless, our understanding of mechanisms underlying PC progression (metastasis and CRPC development) remains incomplete.

The chromosomal locus 8q24 is one of the most frequently amplified and altered regions in a variety of cancer types, including ovarian [13], colorectal [14–17], breast [18–22], prostate [23–28], and others. The 8q24.21 locus is particularly attractive, in part attributable to its containment of the most commonly amplified gene in cancer, MYC. As expected, the 8q24.21 region is amplified in PCs [29, 30]. Variations in the locus associate with an increased risk of PC development [15, 24–26, 31, 32]. The 8q24.21 locus contains a 1.2Mb gene desert bounded by FAM84B and MYC at the centromeric and telomeric end, respectively [15, 21, 24, 25, 27]. A pseudogene of POU5F1, POU5F1P1/POU5F1B, lies within this gene desert [33] and elevations of POU5F1P1 at both the mRNA and protein levels were observed in PCs [34]. While alterations in MYC is well demonstrated during PC tumorigenesis [29, 35], changes in this locus for FAM84B in PCs remain unclear.

We demonstrate for the first time that the FAM84B gene is amplified and expression is upregulated during prostate tumorigenesis, and follows PC progression. FAM84B upregulation was observed in DU145 cell-derived prostate cancer stem-like cells (PCSLCs) in comparison to non-PCSLCs and in prostate carcinoma compared to normal prostate tissues. The upregulation and FAM84B gene amplifications are particularly clear in CRPCs.

## RESULTS

### Increased levels of FAM84B in PCSLCs

**Figure 1:**
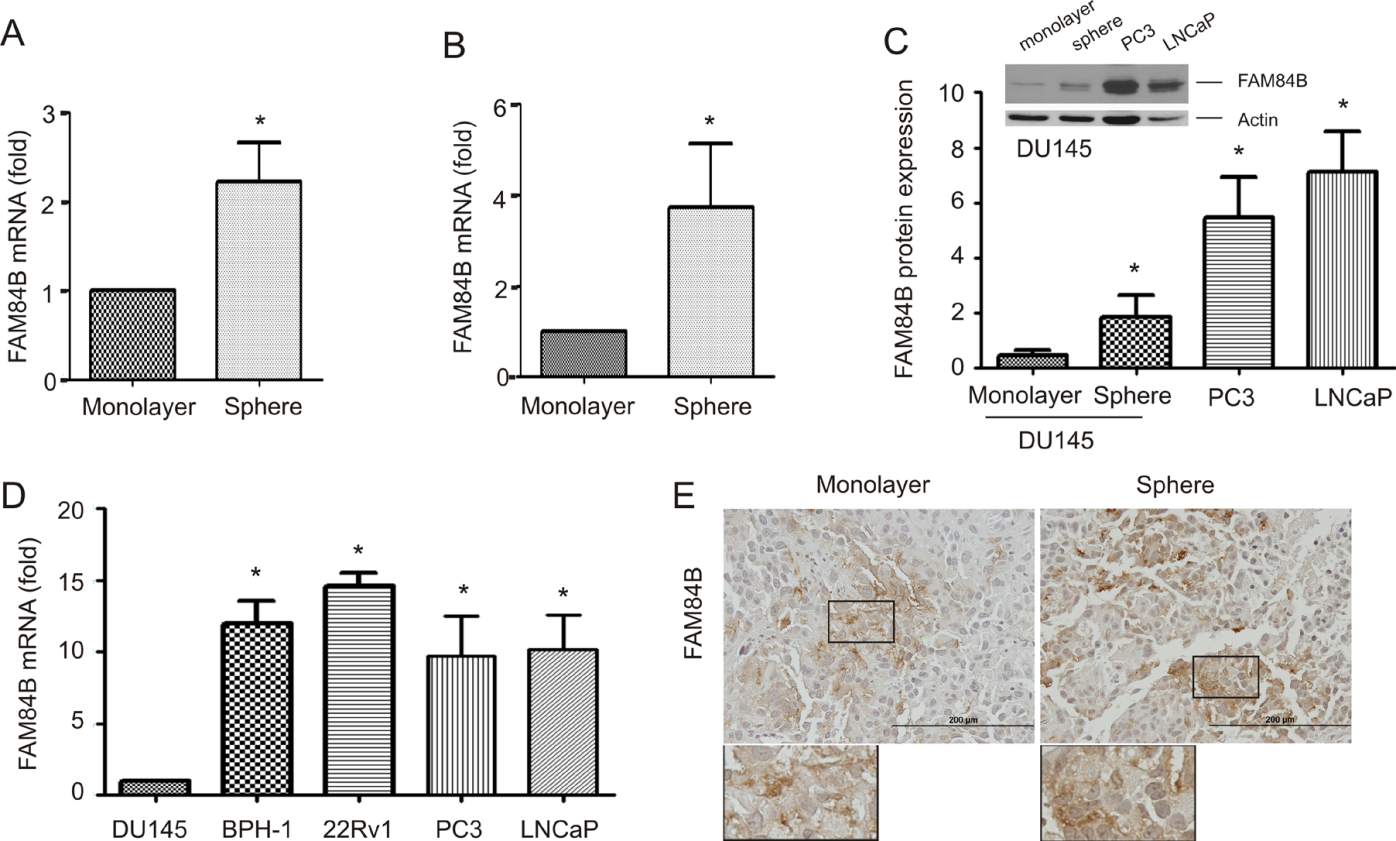
Upregulation of FAM84B in prostate cancer stem-like cells (PCSLCs) (**A**) Affymetrix profiling gene expression in DU145 monolayer and sphere cells. The profiling was carried out three times with duplicates for each repeat. FAM84B mRNA is expressed as fold change compared to monolayer cells. Mean±SD (standard deviation) are graphed. Statistical analysis was performed using 2-tailed Student's *t*-test. (**B**, **D**) Real-time PCR analysis of FAM84B mRNA in DU145 monolayer, DU145 sphere cells (PCSLCs), and the indicated PC lines. β-actin was used as an internal control. Experiments were repeated three times. FAM84B mRNA abundance is graphed as a fold change to DU145 monolayer cells; mean ± SD are graphed. *:*p* < 0.05 by a 2-tailed Student's *t*-test. (**C**) Western blot examination of FAM84B protein expression in DU145 monolayer and sphere, PC3, and LNCaP cells. Experiments were repeated three times; typical images from a single repeat are shown (inset). FAM84B protein levels were normalized to actin; mean±SD are graphed. *p < 0.05 by a 2-tailed Student's *t*-test in comparison to DU145 monolayer cells. (**E**) FAM84B protein expression in xenograft tumors produced by either DU145 monolayer or sphere cells. The overall patterns of FAM84B expression at low magnification are presented in Supplementary Figure 2.

*MYC* resides in 8q24.21 and is one of four genes that were initially identified to program fibroblast cells into induced pluripotent stem cells (iPSCs) [36]. Considering *MYC* and *FAM84B* are located at either end of the 8q24.21 gene desert (Supplementary Figure 1), a potential association of FAM84B with PCSLCs was examined. We have recently established PCSLCs grown as spheres in suspension from DU145 monolayer cells [37]. To examine FAM84B expression in spheres cells versus monolayer cells, we analyzed their gene expression profiles by conducting three separate affymetrix microarrays. A significant increase in FAM84B mRNA in spheres was observed (Figure 1A), while no changes in both MYC and POU5F1P1 were detected, suggesting that the upregulation of FAM84B was specific. This elevation was confirmed by real time PCR and Western blot (Figure 1B, 1C). In comparison to DU145 cells, PC3 and LNCaP cells express higher levels of FAM84B at both the protein and mRNA levels (Figure 1C, 1D). We also noticed an elevation of FAM84B mRNA in another androgen-sensitive PC line (22Rv1) and an immortalized prostate epithelial cell line (BPH-1) compared to DU145 cells (Figure 1D). The underlying reason for increased levels of FAM84B in immortalized BPH-1 cells as well as androgen sensitive LNCaP and 22Rv1 cells compared to more aggressive androgen resistant DU145 cells remains unclear (see Discussion for details). To further examine PCSLC-associated FAM84B upregulation, we analyzed its expression in xenograft tumors produced by either DU145 monolayer or sphere cells. Despite a significantly higher level of FAM84B expression in sphere cells *in vitro* (Figure 1A. 1B), there were no clear differences in FAM84B protein expression between the two types of xenograft tumors (Figure 1E, Supplementary Figure 2). These observations suggest that the mechanisms underlying FAM84B upregulation in DU145 PCSLCs are attributable to epigenetic changes instead of gene amplification, which may contribute to only FAM84B's increased expression among the three genes (*FAM84B*, *POU5F1P1*, and *MYC*) in the 8q24.21 gene desert (Supplementary Figure 1).

### FAM84B upregulation associates with PC tumorigenesis

**Figure 2:**
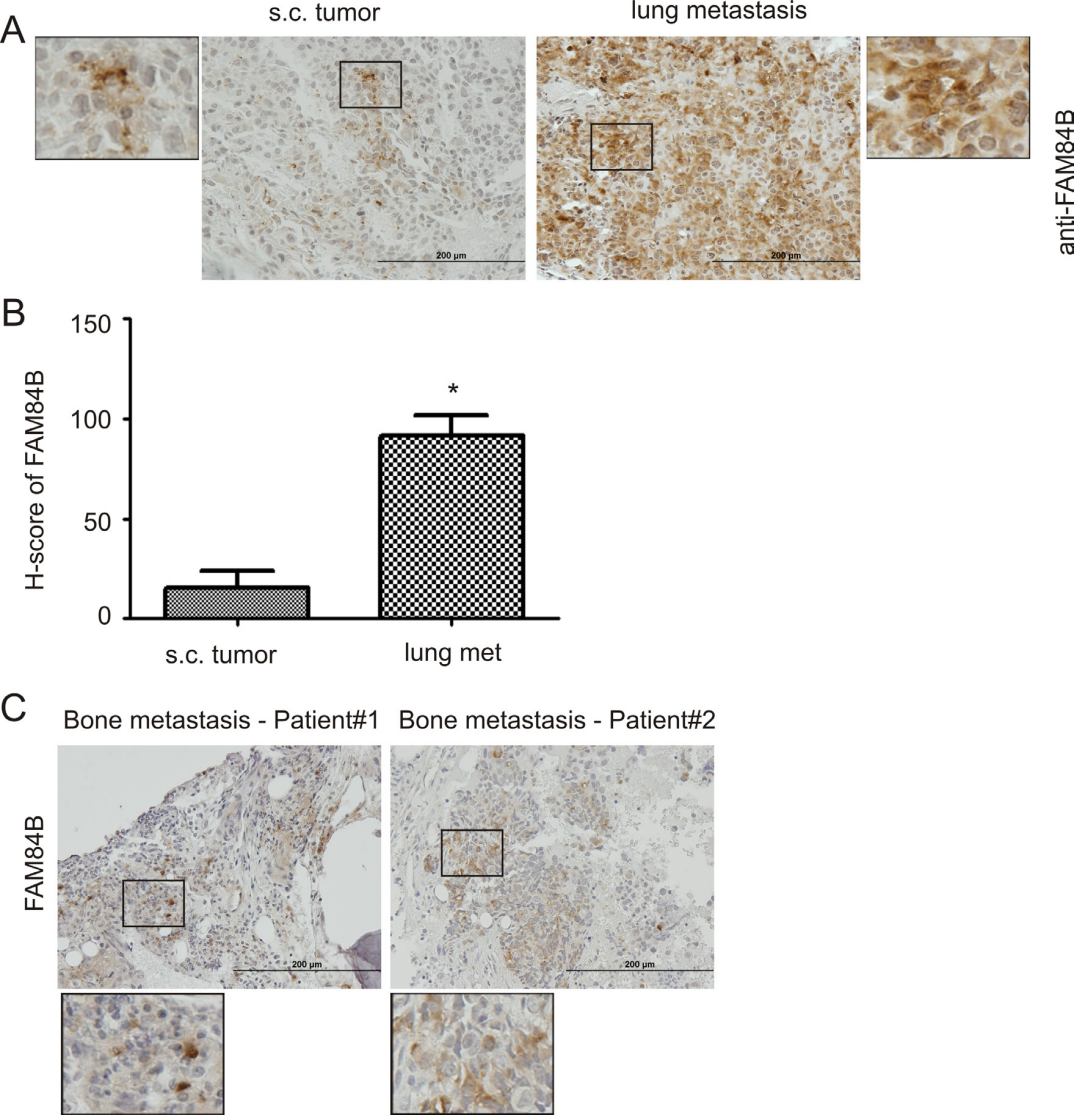
Upregulation of FAM84B in metastatic PC DU145 subcutaneous (s.c.) xenograft tumors and lung metastasis were produced in NOD/SCID mice (5 mice per group). (**A**) Typical IHC staining for FAM84B in s.c. tumors and lung metastasis (see Supplementary Figure 3A for overall staining). The indicated regions are enlarged three fold and placed on the side of the original panel. (**B**) IHC staining was quantified through ImageScope software. Average HScores±SDs are graphed. *p < 0.05 by a 2-tailed Student's *t*-test. (**C**) IHC staining for FAM84B was performed on four human bone metastases; two tumors with positive staining are shown here (see Supplementary Figure 3B for additional images). Indicated regions are enlarged 2.5 fold and placed beneath the original panel.

We have previously shown that DU145 sphere cells (PCSLCs) display a 100-fold higher tumorigenic ability [37]. While we did not detect elevated FAM84B expression in sphere cell-derived xenograft tumors (see Discussion for details) (Figure 1E, Supplementary Figure 2), it remains possible that FAM84B upregulation associates with PC tumorigenesis and/or progression. To investigate this scenario, we produced subcutaneous (s.c.) xenograft tumors and lung metastasis (via tail vein injection) using DU145 monolayer cells [38]. FAM84B expression was heterogeneously detected in both tumor types (Supplementary Figure 3A); FAM84B is largely a cytosolic protein (Figure 2A, enlarged images), and is clearly expressed at an elevated level in lung metastasis (Figure 2A, 2B; Supplementary Figure 3A). Interestingly, the staining showed higher levels of expression in the tumor edges adjacent to normal mouse lung tissue (Supplementary Figure 3A), suggesting a role of FAM84B in facilitating PC metastasis. This concept is supported by our demonstration of FAM84B expression in 2 out of 4 human PC bone metastases (Figure 2C, Supplementary Figure 3B). Collectively, these observations support an association of FAM84B upregulation with PC progression.

To further investigate this association, we determined FAM84B mRNA levels in 7 pairs of PC and benign prostate tissues using Nanostring technology. The PC tissues contained 60–80% cancer tissue. This collection of samples revealed PTEN reductions and ERG increases (indicative of TMPRSS2-ERG fusion) in 5/7 of the PC cases (Table 1), which validated these tissues for analysis of PC-associated gene expression. Impressively, an elevation of FAM84B mRNA was detected in all carcinomas compared to the matched non-tumor prostate tissues (Table 1; *p* = 0.002 by a 2-tailed Student's *t*-test), supporting FAM84B upregulation in PCs.

**Table 1:** Nanostring analysis of gene expression in primary prostate cancer tissues

Genes	P1^a^	P2^a^	P3^a^	P4^a^	P5^a^	P6^a^	P7^a^
FAM84B	+1.3	+3	+1.2	+2.6	+1.6	+2.7	+2.1
TMPRSS2-ERG**^b^**	+16.2	+30.2	N^c^	N	+17	+25.3	+27.3
PTEN**^b^**	−1.4	−1.4	N	N	−1.4	−1.3	−2.6

^a^:patients 1-3 (GS6), patients 4-6 (GS7, 4+3 for P4,5, and 3+4 for P6), and P7 (GS4+4).

^b^:TRPRSS2-ERG and PTEN were used as positive controls for upregulated and downregulated genes in PC. downregulated or upregulated genes.

^c^:no downregulation or upregulation.

**Figure 3:**
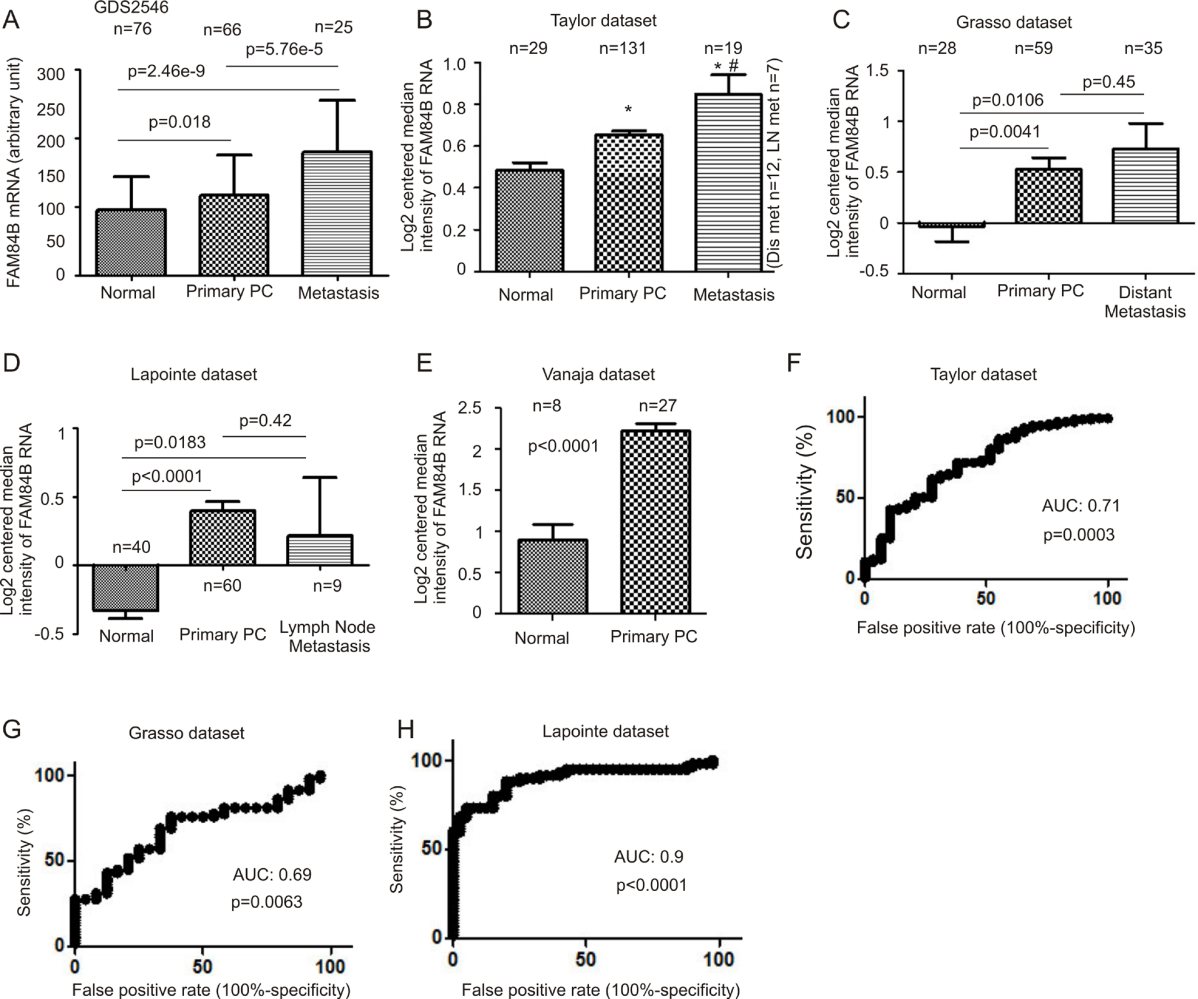
FAM84B upregulation associates with PC tumorigenesis Data were extracted from GDS2546 (Gene Expression Omnibus) (**A**), and Oncomine^TM^ (Compendia Bioscience, Ann Arbor, MI) datasets Taylor (**B**), Grasso (**C**), Lapointe (**D**), and Vanaja (**E**) for analysis of changes in FAM84B mRNA. Mean ± SD are graphed. *p < 0.05 by a 2-tailed Student's *t*-test in comparison to Normal; ^#^*p* < 0.05 in comparison to primary PC (2-tailed Student's *t*-test). (**F**–**H**) A receiver-operating characteristic (ROC) curve of primary prostate tumors versus metastatic PCs was derived from the indicated datasets. AUC: area under the curve.

To further examine this concept, we analyzed gene expression data available from three major databases; Gene Expression Omnibus (GEO) (http://www.ncbi.nlm.nih.gov/geo/) [39], Oncomine^TM^ (Compendia Bioscience, Ann Arbor, MI), and cBioPortal [40, 41] (http://www.cbioportal.org/index.do). We extracted FAM84B mRNA expression data from GSD2546 (dataset2546) (http://www.ncbi.nlm.nih.gov/sites/GDSbrowser) in GEO [42, 43] (Supplementary Figure 4). The dataset contained 17 normal prostate, 59 tumor adjacent normal prostate tissues (*n* = 76), 66 primary PCs, and 25 metastases (lymph node, liver, kidney, and others) (Supplementary Figure 4). A significant increase in FAM84B mRNA can be demonstrated following PC progression from normal prostate tissues to carcinoma and to metastasis (Figure 3A). Elevated FAM84B mRNA in primary PCs over normal prostate tissues was also observed using the FAM84B mRNA data extracted from the Taylor, Grasso, Lapointe, and Vanaja datasets [44] (Figure 3B–3E) within the Oncomine^TM^ database [45–47]. While metastatic PCs expressed higher levels of FAM84B than the normal prostate tissues (Figure 3B–3D), a significant increase in metastases over primary tumors can only be demonstrated in the Taylor dataset (Figure 3B). Together the five datasets contain 181 normal prostates and 343 primary PCs (Figure 3A–3E), and validate the association of FAM84B upregulation with prostate tumorigenesis. This concept is further supported by their respective receiver-operating characteristic (ROC) curves that separate normal prostates from primary PCs via an area under curve (AUC) value ranging from 0.69 to 0.9 (Figure 3F–3H). Nonetheless, the concept of whether FAM84B upregulation correlates with PC progression to metastatic disease warrants further investigations.

**Figure 4:**
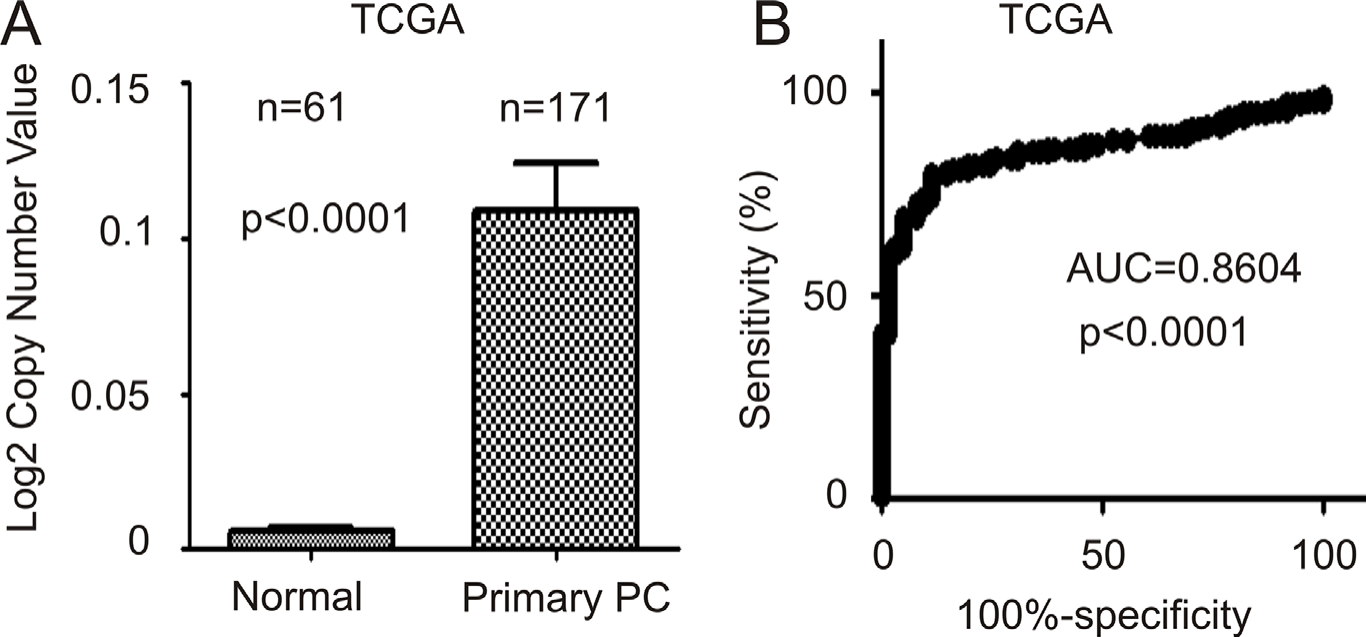
FAM84B gene amplification in prostate tumors FAM84B gene copy variation data were extracted from the TCGA dataset within the Oncomine^TM^ database. (**A**) Mean ± SD are graphed. Statistical analysis was performed using 2-tailed Student *t*-test. (**B**) A ROC curve of primary PC versus normal prostate tissues was calculated.

Higher levels of FAM84B in primary PCs may be in part attributable to increases in gene copy number (GCN). In an analysis of FAM84B GCN data extracted from the TCGA dataset within Oncomine^TM^, FAM84B GCN is significantly increased in 171 primary PCs compared to 61 normal prostate tissues (Figure 4A). The differences separate the two groups with an AUC value of 0.86 (*p* < 0.0001) (Figure 4B).

### FAM84B expression does not correlate with primary PC progression

**Figure 5:**
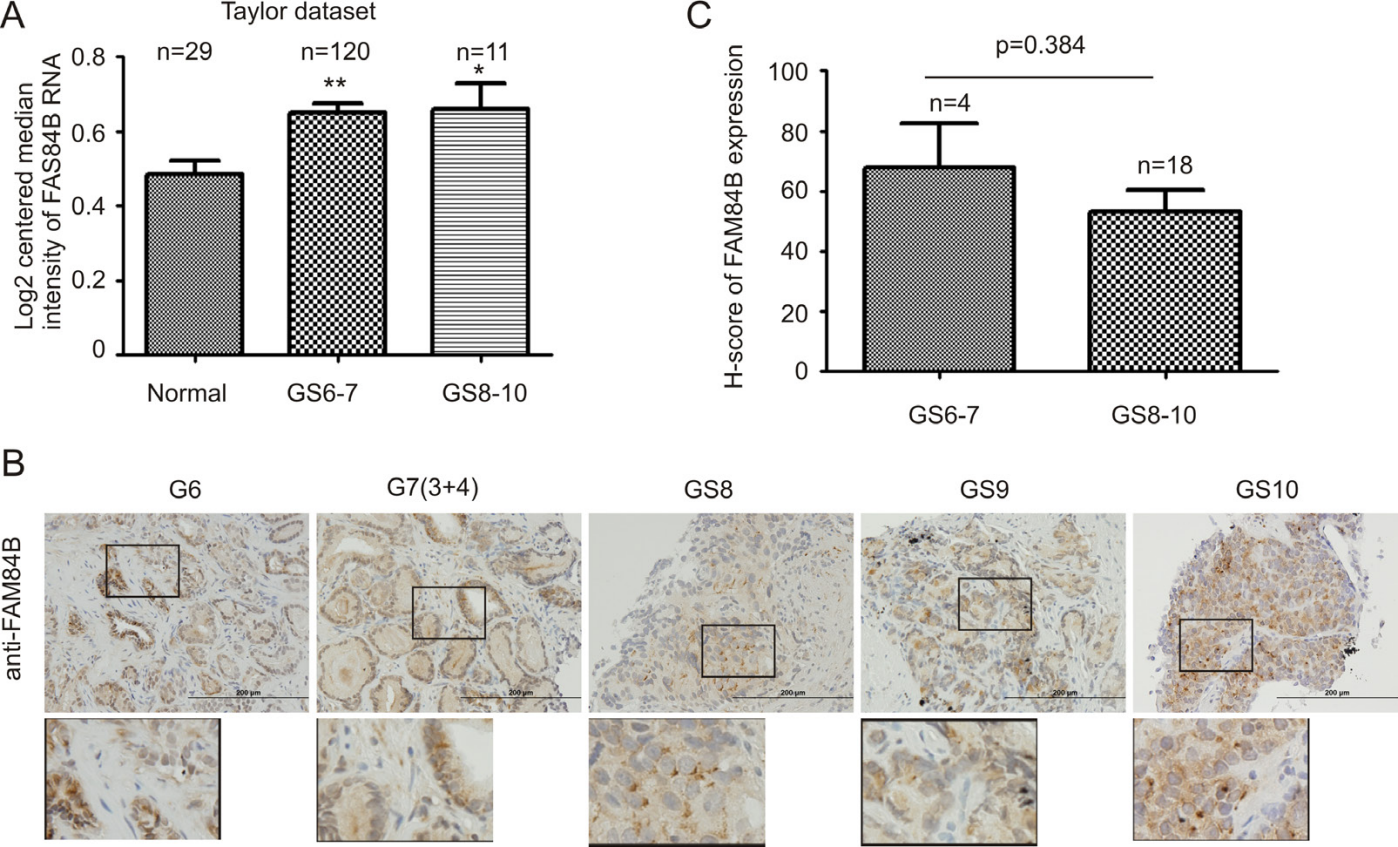
FAM84B upregulation does not associate with Gleason score (GS) advancement (**A**) FAM84B mRNA expression data were extracted from the Taylor dataset in the Oncomine^TM^ database, and were analyzed as low grade (GS6-7) or high grade (GS8-10) PCs. *p < 0.05 and **p < 0.01 in comparison to normal prostate tissues (2-tailed Student's *t*-test). (**B**) IHC staining for FAM84B in 22 primary PC tissues (Supplementary Table 1). Typical images for tumors with the indicated GS are shown. Scale bars represent 200 μm (See Supplementary Figure 5 for additional images). (**C**) IHC staining was quantified through ImageScope software. Average HScores ± SDs are graphed (for detailed scores, See Supplementary Table 1). Statistical analyses were performed using Student's *t*-test.

Primary PCs can be divided into and analyzed as low grade (Gleason score 6–7/GS6-7) and high grade (GS8-10) tumors [48]. Data extracted from the Taylor dataset [45] indicated FAM84B mRNA levels in both low and high grade PCs are significantly higher than in normal prostates (Figure 5A). Although the number of cases with high grade PCs was limited, high grade PCs do not seem to display higher levels of FAM84B than low grade PCs (Figure 5A). We further examined FAM84B protein expression via immunohistochemistry in our limited patient cohort consisting of 4 low and 18 high grade prostate carcinomas (Supplementary Table 1). The FAM84B protein was clearly detected in low and high grade PCs (Figure 5B). However, we could not demonstrate higher levels of FAM84B expression in high grade PCs (Figure 5C). While FAM84B is largely a cytosolic protein in PC cells (Figure 5B), nuclear staining was also observed in high grade cases (Supplementary Figure 5).

### FAM84B upregulation correlates with CRPC development

A critical form of PC progression is the development of CRPC. To examine whether FAM84B upregulation associates with this, we determined FAM84B expression *in vitro* and *in vivo* under androgen deprivation conditions. Androgen free media for 24 hours robustly reduced prostate specific antigen (PSA) expression in LNCaP cells as expected (Supplementary Figure 6A), however, FAM84B mRNA levels were only slightly reduced (Supplementary Figure 6B). This suggests that FAM84B transcription is not directly controlled by AR signaling.

**Figure 6:**
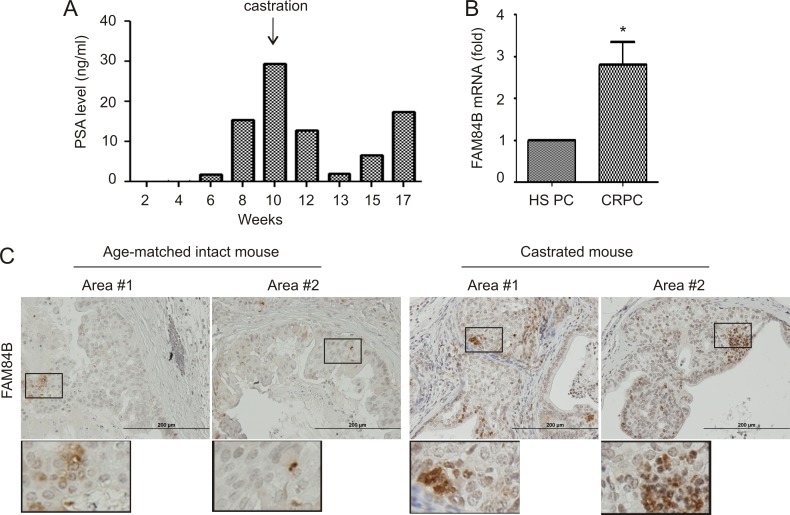
Alterations of FAM84B expression in animal models of castration resistant PC (**A**) PSA levels in NOD/SCID mice (*n* = 3) bearing LNCaP cell-derived xenograft tumors prior to and after castration; a typical pattern from a single mouse is shown. (**B**) Real time PCR analysis of FAM84B mRNA in hormone sensitive (HS) and castration resistant PC (LNCaP xenograft tumors). *p < 0.05 by a 2-tailed Student's *t*-test in comparison to hormone naive xenograft tumors. HS PC: hormone sensitive prostate cancer. (**C**) IHC staining of FAM84B in prostate tumors produced in intact and castrated *PTEN*^−/−^ mice (*n* = 3; see Supplementary Figure 7 for the production of prostate specific PTEN^−/−^ mice). Indicated regions are enlarged 2.5 fold and placed beneath the original panel.

To further investigate FAM84B expression in the process of CRPC development, we generated xenograft tumors using LNCaP cells in intact and castrated NOD/SCID mice (Figure 6A). Castration delayed tumor growth, which was followed by the generation of castration resistant tumors as indicated by changes in PSA levels following castration (Figure 6A). In comparison to xenograft tumors in intact mice, castration resistant tumors demonstrated increases in FAM84B mRNA expression (Figure 6B).

We also examined FAM84B expression in PCs generated in prostate-specific *PTEN^−/−^* mice with and without castration. Prostate specific *PTEN^−/−^* mice were castrated at 23 weeks and euthanized after 13 weeks, and presented with CRPC (Supplementary Figure 7A, 7B). In comparison to hormone naive tumors, prostate tumors produced in castrated *PTEN^−/−^* mice exhibited an elevated level of FAM84B protein expression (Figure 6C). Interestingly, FAM84B is heterogeneously expressed and the positive cells can be clearly detected as cell clusters particularly in castrated mice (Figure 6C; Supplementary Figure 7C), suggesting that FAM84B-positive cells may contribute to the clonal expansion of CRPC cells. Additionally, they were located at the edges of tumor adjacent to normal prostate tissue (Supplementary Figure 7C, image #3). Taken together, the above observations support a correlation between FAM84B upregulation and CRPC development.

To continue examining this correlation, we extracted FAM84B mRNA information from cBioPortal datasets and performed analyses using tools provided on the website [40, 41]. There are currently 5 datasets containing mRNA data in cBioPortal, which cover primary prostate tumors and metastatic castration resistant prostate cancers (mCRPCs) (cBioPortal/ http://www.cbioportal.org/index.do) [45, 49–52]. In two datasets with 549 primary PCs, increases in FAM84B mRNA were detected in 12% (11–13%) of PCs (Table 2); in comparison, FAM84B upregulation occurred in 18% (9–27%) of mCRPCs in three datasets consisting of 412 mCRPCs (Table 2). The differences in the distribution of tumors with FAM84B upregulation in primary PCs and mCRPCs are statistically significant (*p* < 0.025, Table 2). Collectively, our comprehensive analyses (xenograft tumors, PCs generated in prostate specific *PTEN^−/−^* mice, and primary PCs) strongly support the notion that FAM84B upregulation correlates with CRPC development.

**Table 2:** ^a^ Upregulation of FAM84B mRNA following PC progression

Dataset	PC type	Cases^b^	Upregulation^c^ (%)	Ref
CGARN	Primary PC	333	11% (38/333)	Cell 163, 1011–25, 2015
Taylor et al	Primary PC	216	13% (29/216)	Cancer Cell 18, 11–22, 10
Total		549	12% (67/549)	
Kumar et al	mCRPC^d^	176	9% (16/176)	Nat Med 22, 369–28, 16
Robinson et al	mCRPC	118	22% (26/118)	Cell 161, 1215–28, 2015
Beltran et al	mCRPC	114	27% (31/114)	Nat Med 22, 298–305, 16
Total		412	18% (73/412)*	

a: data were extracted from cBioPortal; b: number of cases; c: upregulation was defined by z-score ≥ 2; d: metastatic castration resistant prostate cancer; *p < 0.025 in comparison to the average rate of FAM84B upreglation by Fisher's Exact Test.

### FAM84B gene amplification correlates with CRPC development

**Figure 7:**
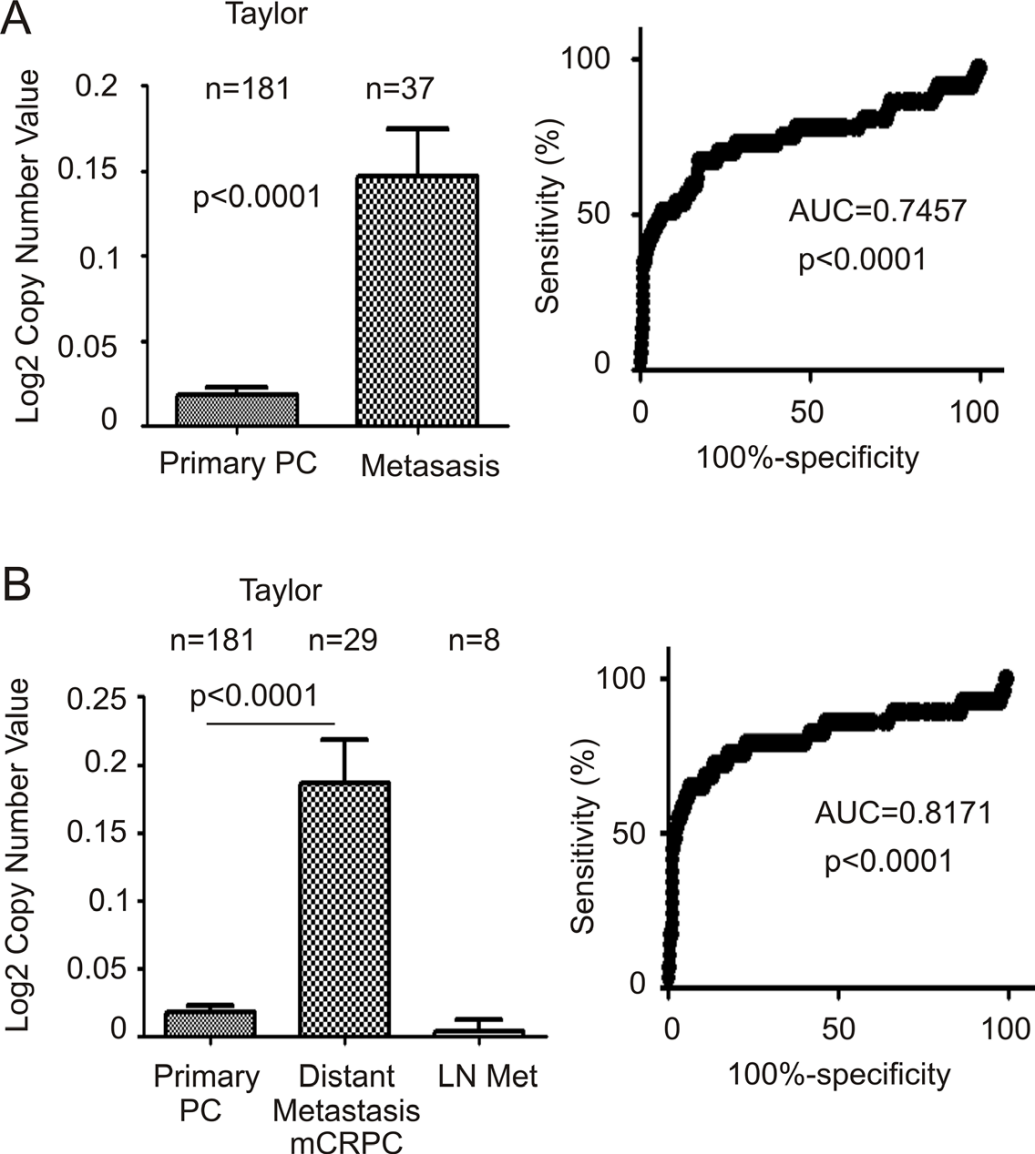
FAM84B is increased in metastatic castration resistant prostate cancer (mCRPC) Data related to FAM84B gene copy number was extracted from the Taylor dataset within Oncomine^TM^ (Compendia Bioscience, Ann Arbor, MI). (**A**) mean ± SD (left panel) and a ROC curve (right panel) of primary versus metastatic PC were calculated and graphed. Statistical analyses were performed using Student's *t*-test. (**B**) The same data were analyzed by separating out distant metastasis (Dis met)/mCRPC from lymph node metastases (LN met). An ROC curve for this arrangement is shown.

We subsequently analyzed FAM84B gene amplification and its association with CRPC. The Taylor dataset within Oncomine^TM^ contains GCN data for 181 primary PCs and 37 metastatic prostate tumors [45]. FAM84B GCN is significantly increased in metastatic PCs compared to primary tumors (Figure 7A, left panel). The metastatic PCs consist of mCRPC and lymph node (LN) metastases [45]; the significant increases in FAM84B GCN observed in the metastatic PC population were attributed to mCRPCs (Figure 7B, left panel). Additionally, the respective ROC curves differentiate primary PCs from mCRPCs (distant metastases) with increased accuracy (AUC = 0.82; Figure 7B, right panel) compared to all metastases including LNs (AUC = 0.75; Figure 7A, right panel).

The association of FAM84B gene amplification was also demonstrated using seven large datasets within the cBioPortal database [45, 46, 50–54] (Table 3). While the FAM84B gene was amplified in 4.8% (0.9–7%) of 546 primary PCs, an average rate of 26% (13–44%) was found in 467 mCRPCs (Table 3, *p* < 0.0001).

**Table 3:** ^a^ FAM84B gene amplification in CRPCs

Dataset	PC type	Cases^b^	Upregulation^c^ (%)	Ref
Barbieri et al	Primary PC	109	0.9% (1/109)	Nat Genet 44, 685–9, 2012
Hieronymus et al	Primary PC	104	2% (2/104)	PNAS 111, 11139–44, 2014
Taylor et al	Primary PC	333	7% (23/333)	Cancer Cell 18, 11–22, 2010
Total		546	4.8% (26/546)	
Kumar et al	mCRPC^c^	149	30% (44/149)	Nat Med 22, 369–28, 16
Robinson et al	mCRPC	150	13% (20/150)	Cell 161, 1215–28, 2015
Grasso et al	mCRPC	61	16% (10/61)	Nature 487, 239–43, 2012
Beltran et al	mCRPC	107	44% (47/107)	Nat Med 22, 298–305, 16
Total		467	26% (121/467)*	

a: data were extracted from cBioPortal.

b: number of cases.

c: metastatic castration resistant prostate cancer.

*p < 0.0001 in comparison to the average rate of FAM84B gene amplification by Fisher's Exact Test.

**Figure 8:**
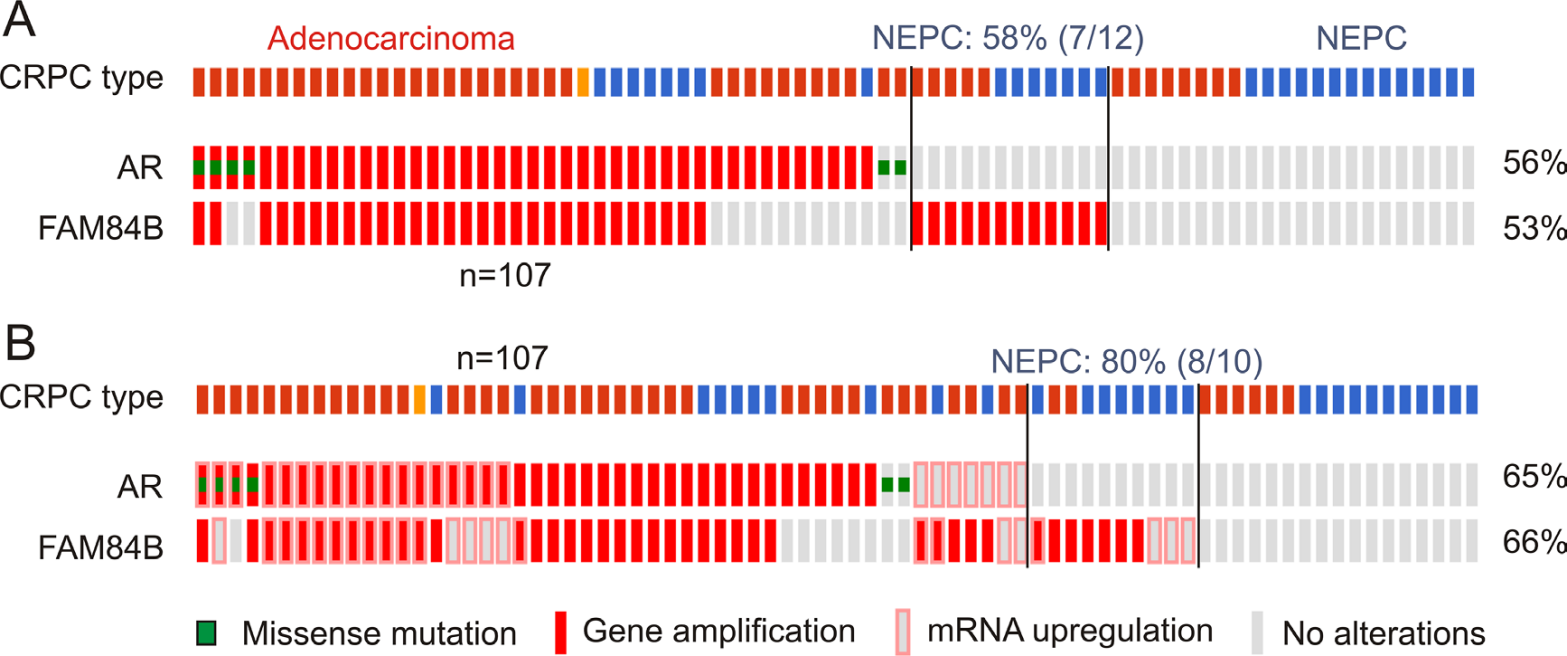
Genomic alterations of the AR and FAM84B genes Data were extracted from the Beltran dataset within the cBioPortal database [40, 41]. The dataset covers 107 CRPCs derived from 77 patients [52]. The indicated genomic alterations without (**A**) and with the respective mRNA upregulations (**B**) in 77 CRPC patients are shown. The percentages of alterations were based on the patient population (*n* = 77). CRPC tumor types are also indicated; red bars: adenocarcinoma, blue bars: neuroendocrine prostate cancer (NEPC), orange bar: adenocarcinoma mixed with NEPC. Symbols for genomic mutations and mRNA upregulation for the AR and FAM84B genes are indicated.

Persistent AR signalling though alterations in AR is known to contribute to CRPC progression [5], suggesting a relationship between genomic changes of the FAM84B and AR genes. In support of this possibility, we obtained evidence for a co-occurrence of AR genomic changes and increased FAM84B GCNs; co-occurrence of the two covers a large proportion of the mCRPCs with FAM84B gene amplification in individual datasets (Figure 8A, Supplementary Figure 8A) [46, 50–52]. The co-occurrence also includes AR and FAM84B mRNA upregulation (Figure 8B, Supplementary Figure 8B), suggesting a role of AR signalling in FAM84B's genomic alterations and upregulation.

These changes in FAM84B also take place independently of AR signalling (Figure 8, Supplementary Figure 8). Intriguingly, in those mCRPCs, 58% (7/12) are the neuroendocrine type (NEPC) (Figure 8A); with FAM84B mRNA upregulation included, the NEPC enrichment reaches 80% (8/10) (Figure 8B), suggesting that CRPCs with changes occurring only in FAM84B are likely NEPCs. In the other three datasets, it would be interesting to examine how many NEPC cases are among the 25 mCRPCs with changes detected only in FAM84B (Supplementary Figure 8).

### FAM84B genomic changes and mRNA upregulation associate with a reduction in disease free survival (DFS)

**Figure 9:**
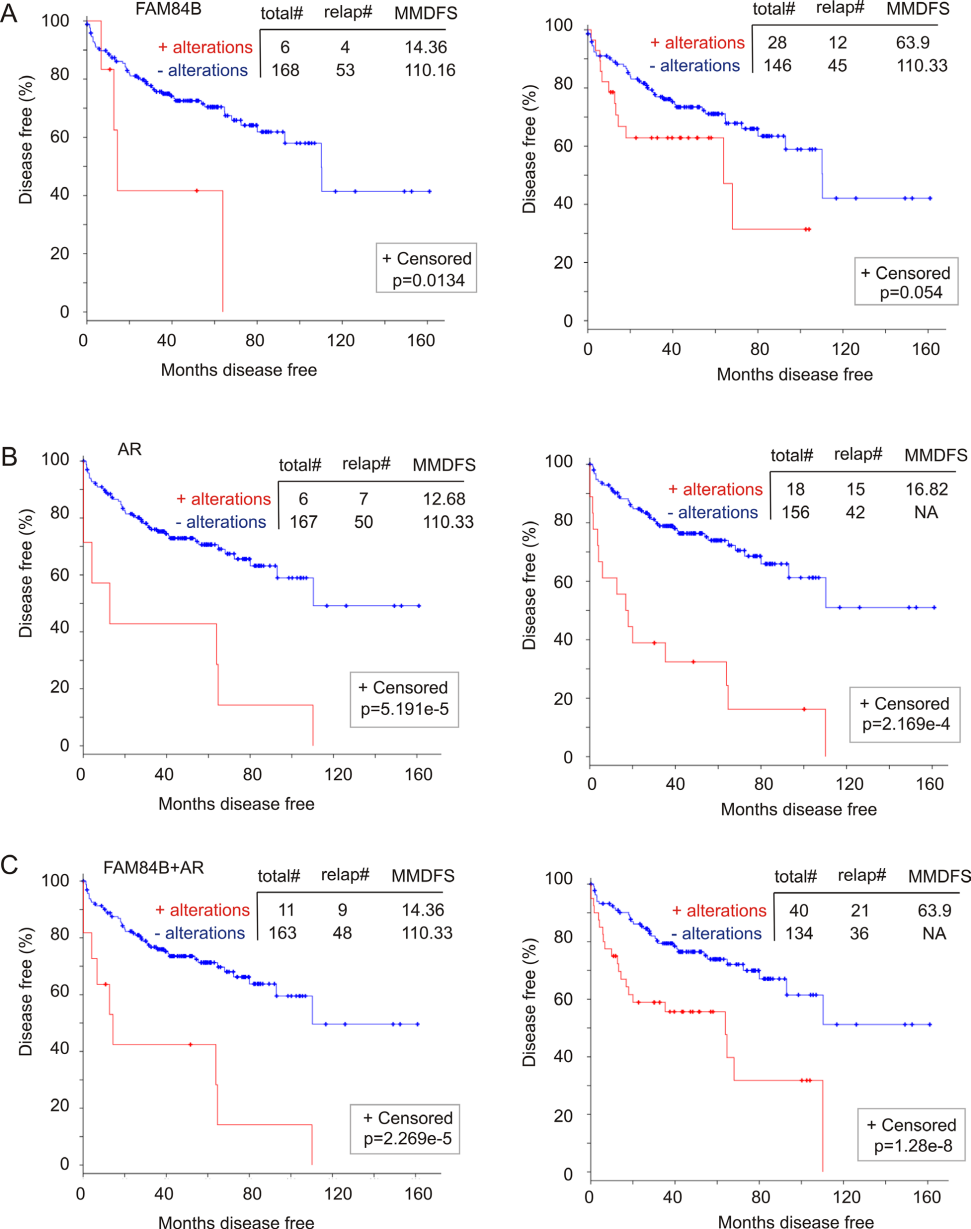
Genomic alterations in the FAM84B gene associate with a reduction in disease free survival (DFS) A dataset of primary PCs [45] (within the cBioPortal database [40, 41]) were used to assess the impact of FAM84B (**A**), AR (**B**), and FAM84B+AR (**C**) genomic changes on DFS without (left panels) and with the respective mRNA upregulation (right panels). The detailed alterations are documented in Supplementary Figure 9. Statistical analysis was performed using Logrank Test. Total#: total number of cases; relap#: number of relapsed cases; MMDFS: median months disease free survival; NA: not available. Censored individuals are indicated; the number of censored individuals is the total individuals minus relapsed patients.

CRPC is a major stage of PC evolution. Our observed correlation of FAM84B changes (genomic alterations and mRNA upregulation) with CRPC development indicates a relationship with PC recurrence (DFS). Of note, by extracting and analyzing genomic and mRNA data from 194 prostate tumors with copy number alterations (CNA) from the “MSKCC, Cancer Cell 2010” dataset [45] within cBioPortal, we were able to show that FAM84B gene amplification alone and with its mRNA upregulation (Supplementary Figure 9A) associates with decreases in DFS (Figure 9A). As expected, similar changes in the AR gene (Supplementary Figure 9B) resulted in a rapid PC recurrence (Figure 9B). Although the combination of changes in AR and FAM84B decreases the power of AR-associated changes in predicting PC recurrence (comparing the respective curves and *p* values in panel B and C, Figure 9), the combination covers more recurrent tumors; 7 for AR versus 9 for AR+FAM84B (left panels of Figure 9B, 9C) and 15 for AR vs 21 for AR+FAM84B (right panels of Figure 9B, 9C). These analyses thus support the notion that genomic changes and mRNA upregulation in FAM84B contribute to AR-associated reduction in DFS, a concept that is in line with the aforementioned changes in FAM84B occurring concurrently and independently of the respective changes in AR in this patient cohort (Supplementary Figure 9C).

**Figure 10:**
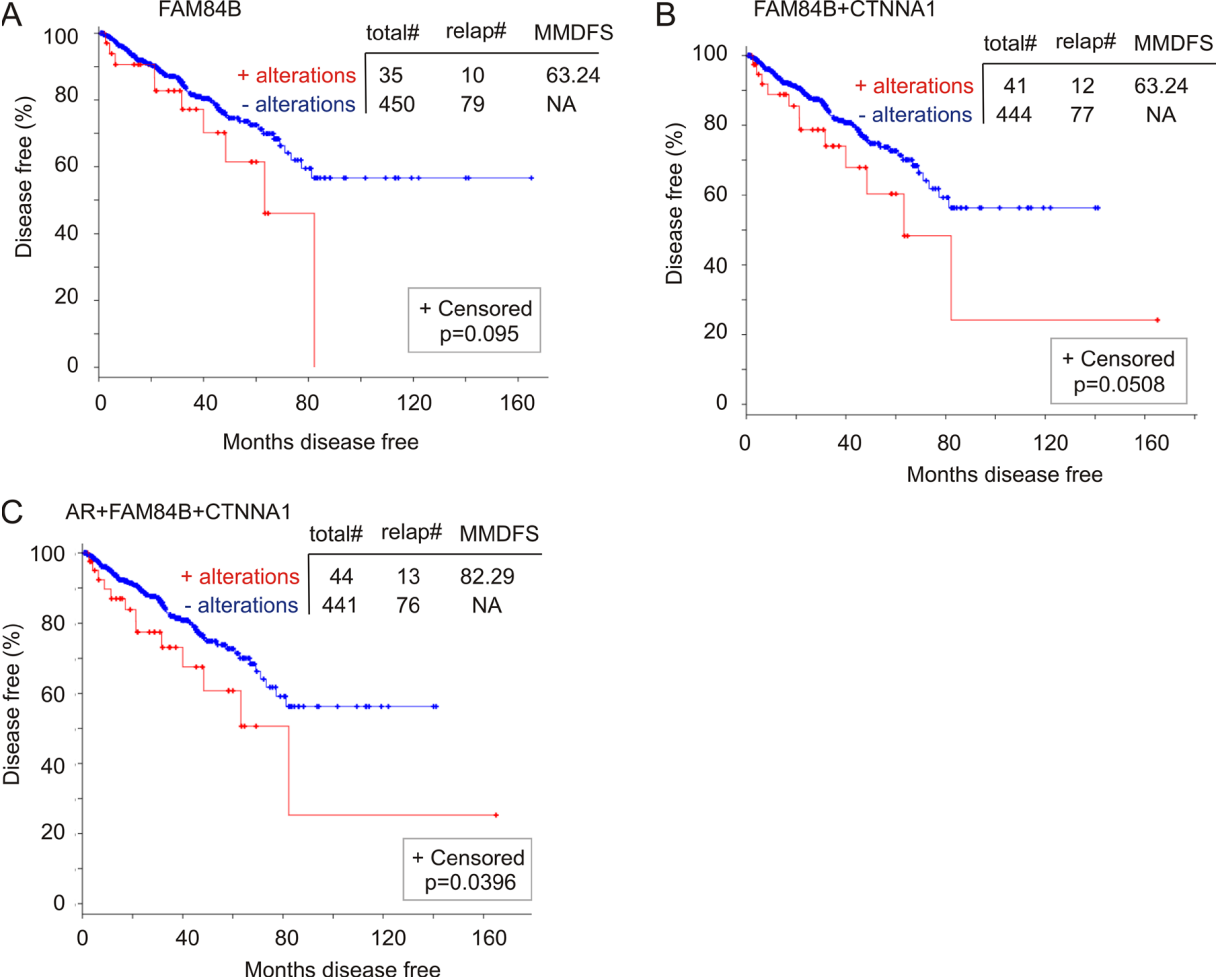
Genomic alterations in the FAM84B network genes associate with a reduction in DFS The TCGA dataset within the cBioPortal database [40, 41] contains 492 primary prostate tumors with copy number variation determined. The detailed genomic alterations in the FAM84B, CTNNA1, and AR genes are presented in Supplementary Figure 10. The effects of these changes with respect to the FAM84B (**A**), FAM84B+CTNNA1 (**B**) or FAM84B+CTNNA1+AR (**C**) are calculated. Statistical analysis was performed using Logrank Test. Total#: total number of cases; relap#: number of relapsed cases; MMDFS: median months disease free survival; NA: not available. Censored individuals are indicated; the number of censored individuals is the total individuals minus relapsed patients.

To further investigate FAM84B gene amplification-associated reductions in DFS, we analyzed a cohort of 492 patients in the TCGA dataset within the cBioPortal database (http://www.cbioportal.org/) in which the PCs have been examined for copy number variations (CNVs) [49]. FAM84B CNVs (largely amplification) were detected in 8% (37/492) of PCs, while AR CNVs occurred in 1% of prostate tumors (Supplementary Figure 10A). In this patient cohort, FAM84B CNVs correlate with a trend of DFS decreases (Figure 10A) and the combination of FAM84B and AR CNVs increased the prediction power (comparing the *p* values in Figure 10A to that in Supplementary Figure 10C). However, the differences do not reach the *p* < 0.05 level of significance (Supplementary Figure 10C).

FAM84B has not been thoroughly studied in the current literature, which results in our lack of knowledge about the proteins or factors that affect FAM84B function. Nonetheless, the cBioPortal database indicates an interactive network between FAM84B and CTNNA1 (α-catenin 1) (Supplementary Figure 11A). In a mCRPC cohort of 107 tumors obtained from 77 patients [52], the CTNNA1 gene was amplified in 21% (16/77) of patients and 15% (16/107) of mCRPCs (Supplementary Figure 11B). Importantly, CTNNA1 gene amplification displays co-occurrence and independence with FAM84B gene amplification (Supplementary Figure 11B). The co-occurrence is also observed with AR and becomes more apparent when mRNA upregulations are included in the comparisons (Supplementary Figure 11C). These observations together with the different chromosomal locations for FAM84B (8q24.21) and CTNNA1 (5q31.2) collectively support a network relationship between the two proteins. Of note, we noticed that although CTNNA1 genomic alterations were detected in 1% of the TCGA cohort of 492 patients (Supplementary Figure 10A), genomic alterations in the network are likely associated with DFS reductions (Figure 10B). Furthermore, the combination of AR genomic changes with those of the network associates with a decline in DFS (Figure 10C). Taken together, evidence supports an association of FAM84B genomic alterations with decreases in DFS in PC patients.

### FAM84B genomic alterations correlate with a reduction in overall survival (OS)

**Figure 11:**
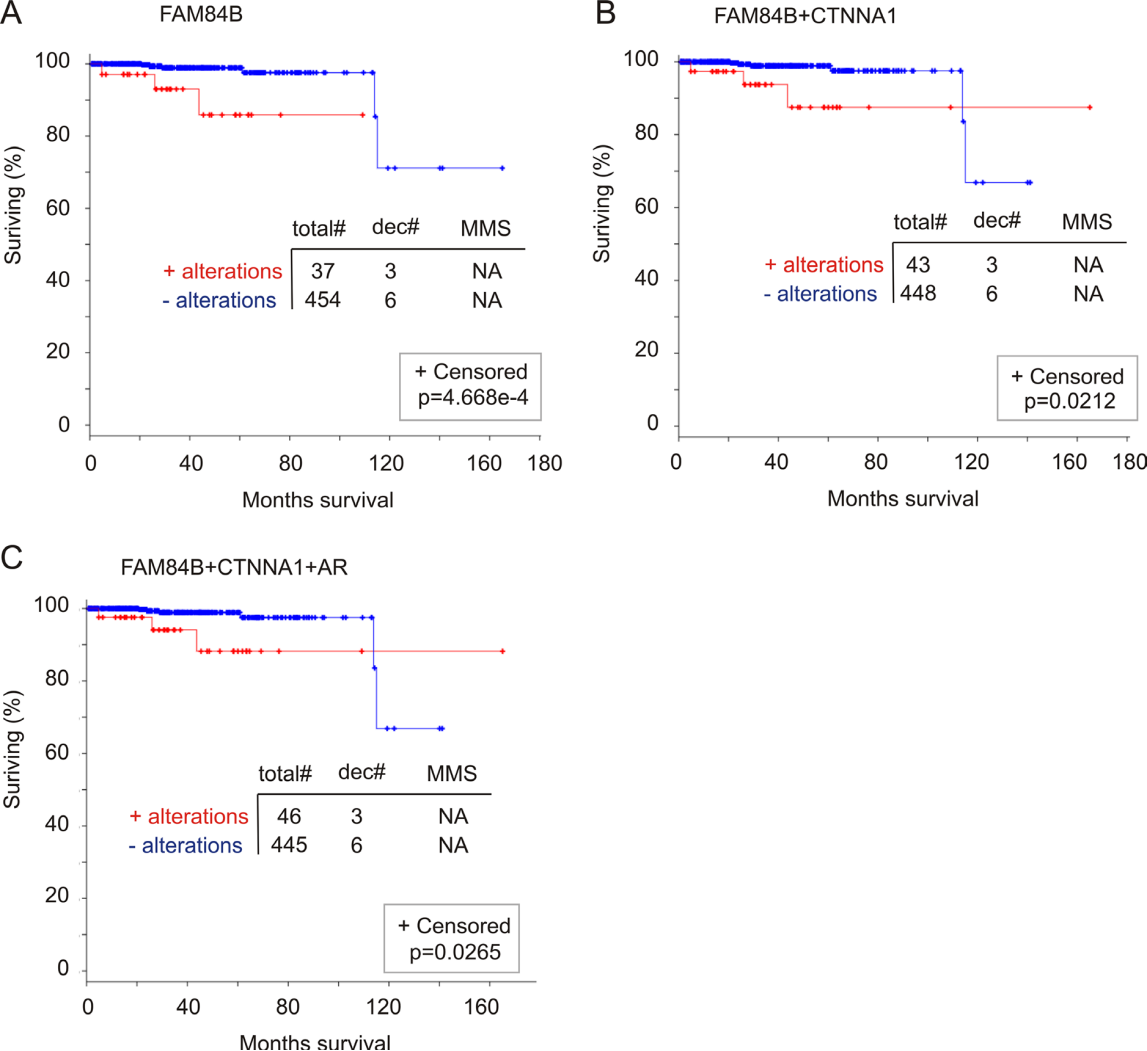
Genomic alterations in the FAM84B gene associate with a reduction in overall survival (OS) The TCGA dataset within the cBioPortal database [40, 41] contains 492 primary prostate tumors with copy number variation determined. The detailed genomic alterations in the FAM84B, CTNNA1, and AR genes are presented in Supplementary Figure 10. The effects of genomic alterations involving the FAM84B (**A**), FAM84B+CTNNA1 (**B**) or FAM84B+CTNNA1+AR (**C**) are calculated. Statistical analysis was performed using Logrank Test. Total#: total number of cases; dec#: number of deceased cases; MMS: median months survival; NA: not available. Censored individuals are indicated; the number of censored individuals is the total individuals minus relapsed patients.

We examined a potential association of FAM84B genomic alterations with OS. Among the 11 datasets related to genomic alteration in PC from cBioPortal [40, 41], two have follow-up data for OS, one for mCRPC patients [46] and another for primary PCs (*n* = 492) with CNVs determined (TCGA, http://www.cbioportal.org/) [49]. Genomic changes in AR, FAM84B, and CTNNA1 individually or in any combination have no relationship with OS in mCRPC patients (data not shown). In the primary PC patient cohort, the 8% of genomic alterations in the FAM84B gene significantly associate with a reduction in OS (Figure 11A). Although the number of cases associated with PC-related fatality is small (*n* = 9), a third (3/9) of these deaths occurred in patients with prostate tumors in which the FAM84B gene has been altered (Figure 11A). Combination with either CTNNA1 or AR does not enhance the association (Figure 11B, 11C). In fact, all fatalities in patients with PC harboring either AR or CTNNA1 genomic alterations were observed in patients with tumors containing FAM84B genomic alterations. Taken together, evidence suggests that genomic alterations in the FAM84B gene correlate with poor prognosis in patients with primary prostate tumors.

## DICUSSION

The chromosome locus 8q24 is one of the most frequently modified loci in cancer and these alterations are associated with the development of PC and other cancer types [27, 33]. The tumorigenic functions conferred by the 8q24 regions can be attributable to the changes in a gene desert at 8q24.21 that is flanked by FAM84B and MYC [27, 28, 33, 55, 56]. While it has been puzzling as to why the non-coding region is frequently targeted during tumorigenesis, a recent development revealed the gene desert as being a regulatory hub in causing genomic alterations in other chromosome loci via physical interaction, including CD96 at 3q13, during prostate tumorigenesis [57]. While this research sheds light on the functional consequences of those alterations in the gene desert, genomic changes of MYC have been well demonstrated in promoting tumorigenesis. However, the association of FAM84B, another major component of the gene desert at the 8q24.21 locus (Supplementary Figure 1), with tumorigenesis remains unclear.

This research is the first attempt to thoroughly investigate the correlation of FAM84B with PC tumorigenesis and progression. By using comprehensive experimental systems involving tissue culture, xenograft tumors, and transgenic mice, by examining alterations in the FAM84B gene and mRNA levels, and by analyzing multiple large datasets, this investigation reports several novel observations: FAM84B upregulation occurs in PCSLCs, FAM84B expression is elevated in primary PCs compared to normal prostate tissues, and there is a significant increase in FAM84B mRNA and gene amplification in CRPC development.

The upregulation of FAM84B could only be detected in DU145-derived PCSLCs *in vitro*. In xenograft tumors produced by PCSLCs and non-PCSLC DU145 cells, elevation of FAM84B expression could not be demonstrated in the prior (Figure 1E, Supplementary Figure 2). This observation in xenograft tumors is likely attributable to PCSLC-mediated tumor regeneration, in which non-PSCLCs are produced. In supporting this possibility, we observed a reduction in PCSLCs self-renewal ability following culture in medium containing 10% fetal bovine serum [37] and also a reduction in PCSLC-associated proteins in xenograft tumors derived from them [38]. However, these tumors do contain PCSLCs which can be re-isolated from the tumor tissue [37]. As a result, we suggest that FAM84B upregulation could not be detected in PCSLC-produced xenograft tumors due to the tumor regeneration process in which the number of PCSLCs are reduced.

The significant upregulation of FAM84B in primary prostate tumors over normal prostate tissues suggests a contribution of FAM84B in PC initiation. However, the mechanisms underlying these changes in FAM84B are likely complex. Nonetheless, our observed FAM84B upregulation in DU145 cell-derived PCSLCs suggests PCSLC-associated plasticity as being a contributor to FAM84B upregulation during PC initiation. This is a possibility that is well in line with the concept of how PCSLCs are also referred to as PC initiating cells. The contribution of PCSLCs to FAM84B upregulation is also supported by the heterogeneous expression of FAM84B protein in xenograft tumors (Figures 1E, 2A) and CRPCs produced in *PTEN^−/−^* mice (Figure 6C). PCSC-mediated FAM84B upregulation during PC tumorigenesis is also in accordance with an elevation of FAM84B mRNA during CRPC development. It is now well-regarded that the plasticity of cancer stem cells drives therapeutic resistance. This plasticity likely contributes to the acquisition of new properties underlying CRPC development. Thus, PCSC-derived plasticity is likely a contributing factor to FAM84B upregulation.

The functional impact of FAM84B on tumorigenesis in general and prostate oncogenesis in particular remains to be explored. Nonetheless, evidence suggests an important role of FAM84B in PC progression. We observed an association of FAM84B genomic alteration and mRNA upregulation with PC recurrence (Figure 9). These results support the theme of this work when they are considered in the context of the research. However, this data should be cautiously interpreted, as the number of patients with alterations is limited (Figure 9A). We also noticed a high level of FAM84B protein expression in the edge regions of DU145 cell-derived lung metastasis and CRPC produced in PTEN deficient mice (Supplementary Figure 3A; Supplementary Figure 7C, image #3), indicating a possible role of FAM84B in mediating PC cell invasion. This concept is supported by recent observations in which knockdown of FAM84B reduced esophageal squamous cell carcinoma cell invasion *in vitro* and from forming xenograft tumors *in vivo* [58, 59]. Whether FAM84B performs a similar function in PC tumorigenesis, particularly in PC metastasis, will be examined in the future.

A possible role of FAM84B in PC progression lies in its potential contributions to CRPC. This possibility is supported by the specific FAM84B upregulation mentioned here (Table 2); this increase occurred uniquely in CRPC and was observed in several independent and large cohorts (Table 2). Furthermore, in our xenograft model for CRPC and transgenic CRPC mouse model, CRPCs exhibited elevated levels of FAM84B compared to hormone naïve tumors (Figure 6). It is thus tempting to propose a role of FAM84B in promoting CRPC development. This hypothesis is supported by a robust elevation in the rate of FAM84B gene amplification from an average 4.8% among 546 primary prostate tumors to 26% in 467 mCRPCs (Table 3). However, it should be stressed that an association of FAM84B upregulation with CRPC is certainly more complex than the aforementioned statements above. For example, FAM84B mRNA is not expressed at higher levels in androgen-resistant DU145 and PC3 cells in comparison to androgen-sensitive LNCaP and 22Rv1 cells or even immortalized human prostate epithelial BPH-1 cells (Figure 1D). The inconsistency between these cell line-based observations and the *in vivo* results (animal models) may be attributable to different systems. Clearly, additional research is required to understand these differences; particularly functional studies need to be performed to evaluate the impact of FAM84B on CRPC development.

While detailed mechanisms leading to FAM84B gene amplification specifically in CRPC remain to be investigated, it is likely that the gene desert, which is bounded by FAM84B at the centromeric side, plays a role. The genomic regulatory elements within the 8q24.21 gene desert were reported to physically associate with FAM84B in a region from 266–440 kb away from the 3′ end of the FAM84B gene in multiple PC cell lines [57]. It is also likely that AR signaling during androgen deprivation also contributes to FAM84B gene amplification. This is in accordance with the importance of persistent AR signalling in CRPC development [5] and in causing genomic instability [60–62]. Furthermore, this possibility is supported by the observed concordance between AR gene amplification and that of FAM84B (Figure 8).

## MATERIALS AND MATHODS

### Cell culture and generation of DU145 spheres (PCSLCs)

LNCaP, PC3, and DU145 cells were purchased from American Type Culture Collection (ATCC), and cultured in RPMI-1640 (LNCaP), F12 (PC3) and MEM (DU145) media supplemented with 10% FBS (Sigma Aldrich) and 1% Penicillin-Streptomycin (Thermo Fisher Scientific). DU145 spheres were generated and cultured according to our published conditions [37]. Briefly, DU145 monolayer cells (non-PCSLCs) were individualized and seeded at a density of 5,000 cells/mL in serum-free (SF) media (3:1 DMEM/F12 mixture) (Thermo Fisher Scientific) containing 0.4% bovine serum albumin (BSA) (Bioshop Canada Inc.) supplemented with 0.2× concentration of B27 minus Vitamin A (Thermo Fisher Scientific) and 10 ng/ml EGF (Sigma Aldrich), in T75 flasks. Typical spheres were formed in 10 to 12 days.

### Collecting primary prostate cancer

Prostate biopsies and radical prostatectomy tissues were obtained at St. Joseph's Hospital in Hamilton, Ontario, Canada under approval from the local Research Ethics Board (REB# 11-3472) and with patient consent.

### Xenograft tumor formation

DU145 (10^6^) or LNCaP cells (5 × 10^6^) were resuspended in 0.1 ml culture media/Matrigel mixture (BD) (1:1 volume), followed by subcutaneous implantation into the flanks of 8 week-old male NOD/SCID mice (The Jackson Laboratory). Tumors were assessed through observation and palpation, and tumor growth was measured weekly using calipers. Tumor volume was determined using the formula V = L × W^2^ × 0.52. Animals were sacrificed once tumors reached a volume ≥ 1000 mm^3^. For the generation of lung metastasis, 10^6^ DU145 cells were resuspended into 0.3 mL of PBS and injected through the tail vein of NOD/SCID mice. Lungs were harvested at 16 weeks post injection. All animal work was carried out according to experimental protocols approved by the McMaster University Animal Research Ethics Board.

### Generation of castration resistant prostate cancer *in vivo*

LNCaP cells (5 × 10^6^) were s.c. implanted into NOD/SCID mice. Tumor growth was monitored by measuring serum PSA levels (PSA kit, Abcam) and physically with calipers as described above. When tumor volume approached 100–200 mm^3^, mice were surgically castrated. Serum PSA was measured prior to and following castration. CRPC was defined when serum PSA rose while tumors continued to grow. Animals were sacrificed once tumors reached a volume ≥ 1000 mm^3^.

PTEN^loxp/loxp^ mice (C;129S4-*Pten^tm1Hwu^*/J) were obtained from The Jackson Laboratory, and PB-Cre4 mice (B6.Cg-Tg(Pbsn-cre)4Prb) were obtained from the NCI Mouse Repository. Prostate specific *PTEN^−/−^* mice were produced by crossing the two strains. Surgical castration was performed when *PTEN^−/−^* mice were 23 weeks old, and observed for 13 weeks before sacrificing.

### Western blot analysis

Cells were lysed in a buffer containing 20 mM Tris (pH 7.4), 150 mM NaCl, 1 mM EDTA, 1 mM EGTA, 1% Triton X-100, 25 mM sodium pyrophosphate, 1 mM NaF, 1 mM β-glycerophosphate, 0.1 mM sodium orthovanadate, 1 mM PMSF, 2 μg/ml leupeptin and 10 μg/ml aprotinin. 50 μg of whole cell lysate was separated on SDS-PAGE gel, and transferred onto Hybond ECL nitrocellulose membranes (Amersham), followed by blocking with 5% skim milk at room temperature for one hour. Primary antibodies were incubated overnight at 4^°^C with agitation, and secondary antibodies incubated for one hour at room temperature. Signals were then developed (ECL Western Blotting Kit, Amersham). Primary antibodies: anti-FAM84B 1:1000 (Proteintech) and anti-Actin 1:1000 (Santa Cruz).

### Quantitative real-time PCR analysis of FAM84B expression

Total RNA was isolated from prostate cancer cell lines and xenograft tissues with Isol-RNA Lysis Reagent (5 PRIME), and reverse transcription was carried out using Superscript III (Thermo Fisher Scientific) according to the manufacturer's instructions. Quantitative real-time PCR was performed using the ABI 7500 Fast Real-Time PCR System (Applied Biosystems) using SYBR-green (Thermo Fisher Scientific). All samples were run in triplicate. FAM84B (Forward): 5′-GACCCAC CTAAGTTACAAGGAAG-3′, FAM84B (Reverse): 5′- GT AGAACACGGAGCATTCCAC-3′. β-Actin (Forward): 5′-TGAAGGTGACAGCAGTCGGT-3′, and β-Actin (Reverse): 5′-TAGAGAGAAGTGGGGTGGCT-3′.

### Immunohistochemistry (IHC)

IHC was performed on 22 paraffin embedded and serially cut prostate cancer tissues obtained from St. Joseph's Hospital, Hamilton, Ontario, Canada, and on the various human xenograft tissues and *PTEN^−/−^* prostates. Slides were deparaffinized in xylene and cleared in an ethanol series. Antigen retrieval was performed in a food steamer for 20 minutes using sodium citrate buffer (pH = 6.0). Tissues were blocked for 1 hour in PBS containing 1% BSA and 10% normal goat serum (Vector Laboratories). FAM84B antibody (1:350, Proteintech) was incubated overnight at 4°C. Secondary antibody biotinylated goat anti-rabbit IgG and Vector ABC reagent (Vector Laboratories) were incubated according to the manufacturer's instructions. Secondary antibody only was used as negative control. Washes were performed with PBS. Chromogenic reaction was carried out with diaminobenzidine (Vector Laboratories), and slides were counterstained with haemotoxylin (Sigma Aldrich). Image analysis was performed using ImageScope software (Leica Microsystems Inc.). Staining intensity values derived from ImageScope were converted to an HScore using the formula [HScore = (% Positive) x (intensity) + 1]. The HScore was normalized through background subtraction and averaged amongst > 5 images per tissue sample.

### Statistical analysis

Statistical analysis is depicted as Mean ± SD or HScores ± SD as stated, and performed using Student's *t*-test where *p* < 0.05 is considered statistically significant. ROC curves were generated using GraphPad Prism 5.0 software. Analysis of data from cBioPortal was performed using Logrank Test.

## SUPPLEMENTARY MATERIALS TABLES AND FIGURES


