# Supplementary material for: Upregulation of FAM84B during prostate cancer progression
Source: Oncotarget. 2017 Feb 7;8(12):19218–35. doi: 10.18632/oncotarget.15168 (PMC5386679; doi:10.18632/oncotarget.15168)
Supplement: Supplementary file 1 [file oncotarget-08-19218-s001.pdf]

Upregulation of FAM84B during prostate cancer progression

Supplementary Materials

Supplementary Table 1: Patient information and FAM84B IHC score

Patient	Age[a]	Gleason Score	Average Score[b]
1	56	3 + 3	97.55 ± 15.03
2	70	3 + 3	64.17 ± 27.47
3	60	3 + 4	80.54 ± 10.14
4	76	4 + 3	28.76 ± 6.28
5	79	4 + 4	52.63 ± 11.52
6	60	4 + 4	41.33 ± 13.89
7	56	4 + 4	30.84 ± 6.95
8	58	4 + 4	34.37 ± 11.77
9	64	4 + 5	40.81 ± 15.75
10	82	4 + 5	45.59 ± 10.39
11	66	4 + 5	12.06 ± 10.83
12	64	4 + 5	107.24 ± 18.83
13	54	4 + 5	19.10 ± 14.31
14	82	4 + 5	88.90 ± 14.25
15	64	4 + 5	24.66 ± 10.23
16	89	5 + 4	11.57 ± 7.13
17	68	5 + 5	94.08 ± 14.55
18	74	5 + 5	65.38 ± 9.42
19	91	5 + 5	61.37 ± 25.27
20	98	5 + 5	75.04 ± 13.86
21	75	5 + 5	72.74 ± 48.46
22	86	5 + 5	81.97 ± 33.09

a:age at diagnosis; b: average score of stain intensity ± SD.

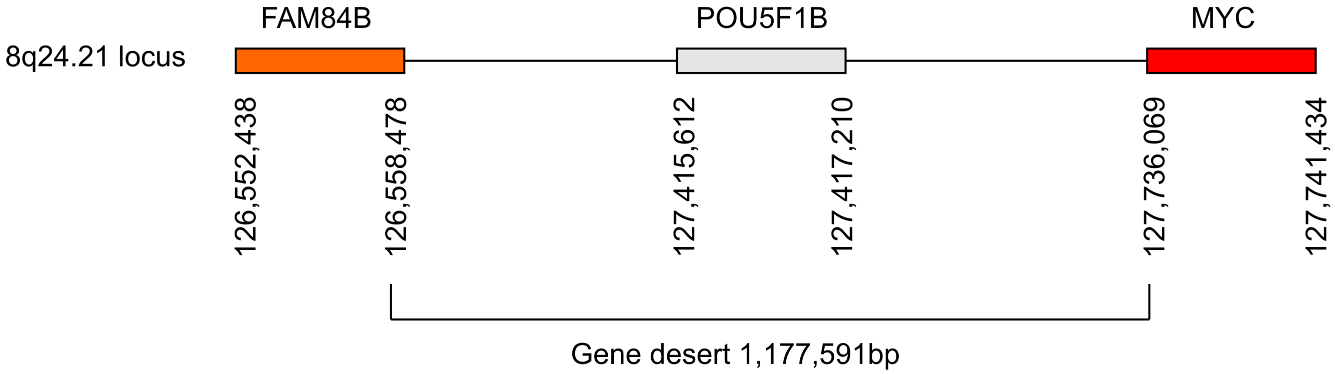

Supplementary Figure 1: A schematic representation of 8q24.21 locus containing the gene desert. The gene location information was obtained from the NCBI Resources (http://www.ncbi.nlm.nih.gov/gene).

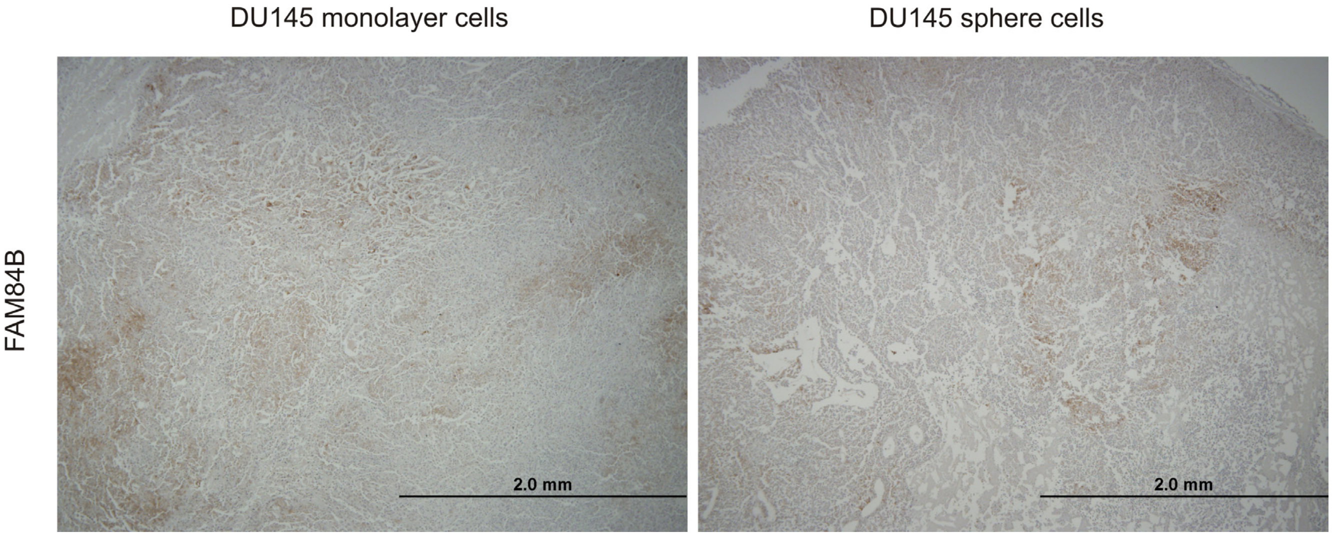

Supplementary Figure 2: FAM84B protein expression in prostate cancer xenograft tumors. Overall immunohistochemistry staining for FAM84B in DU145 cell-derived s.c. xenograft tumors.

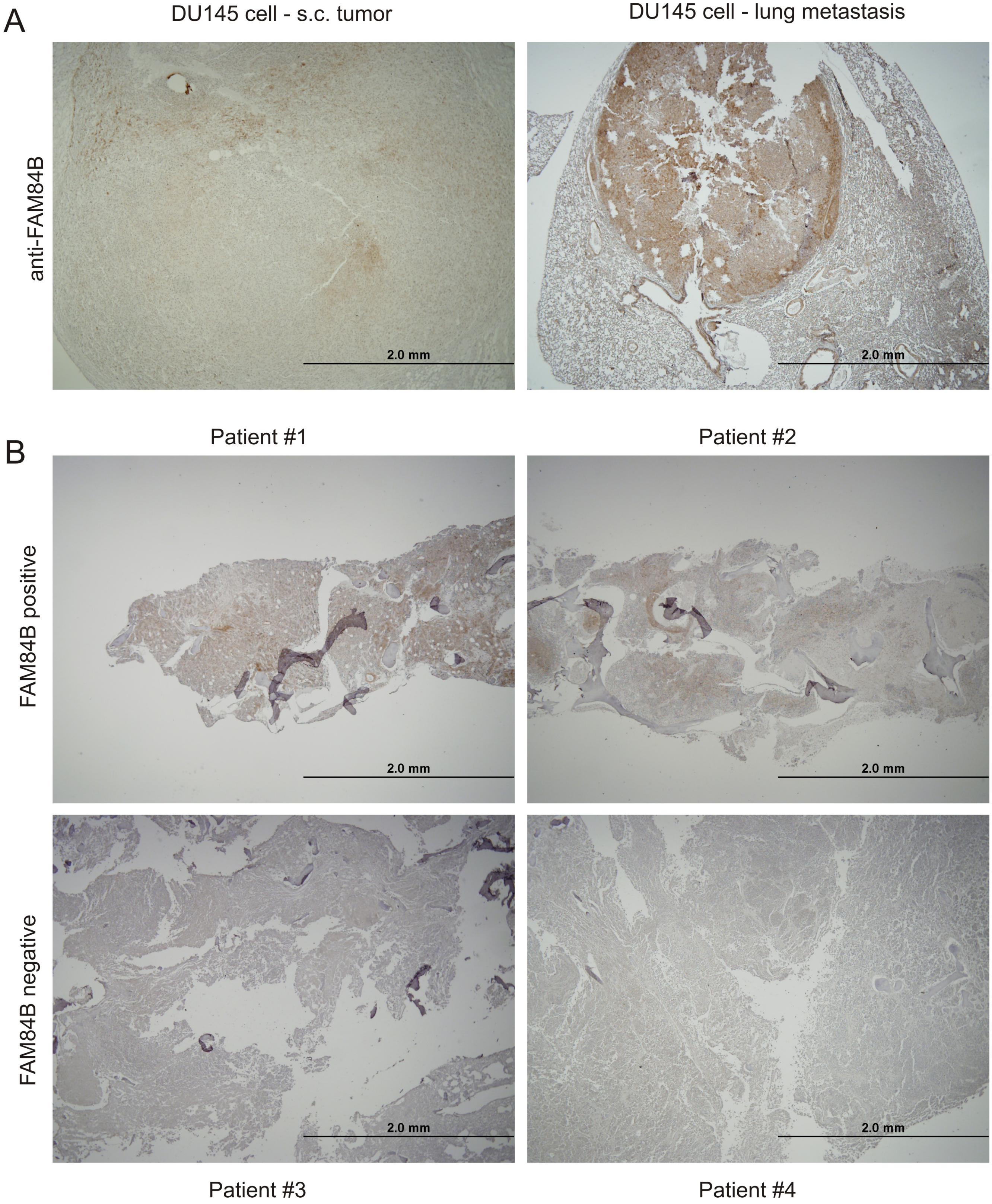

Supplementary Figure 3: FAM84B protein expression in metastatic prostate cancer tissues. Overall immunohistochemistry staining for FAM84B in DU145 cell-derived s.c. xenograft tumor and lung metastases (**A**) and in four human bone metastases (**B**).

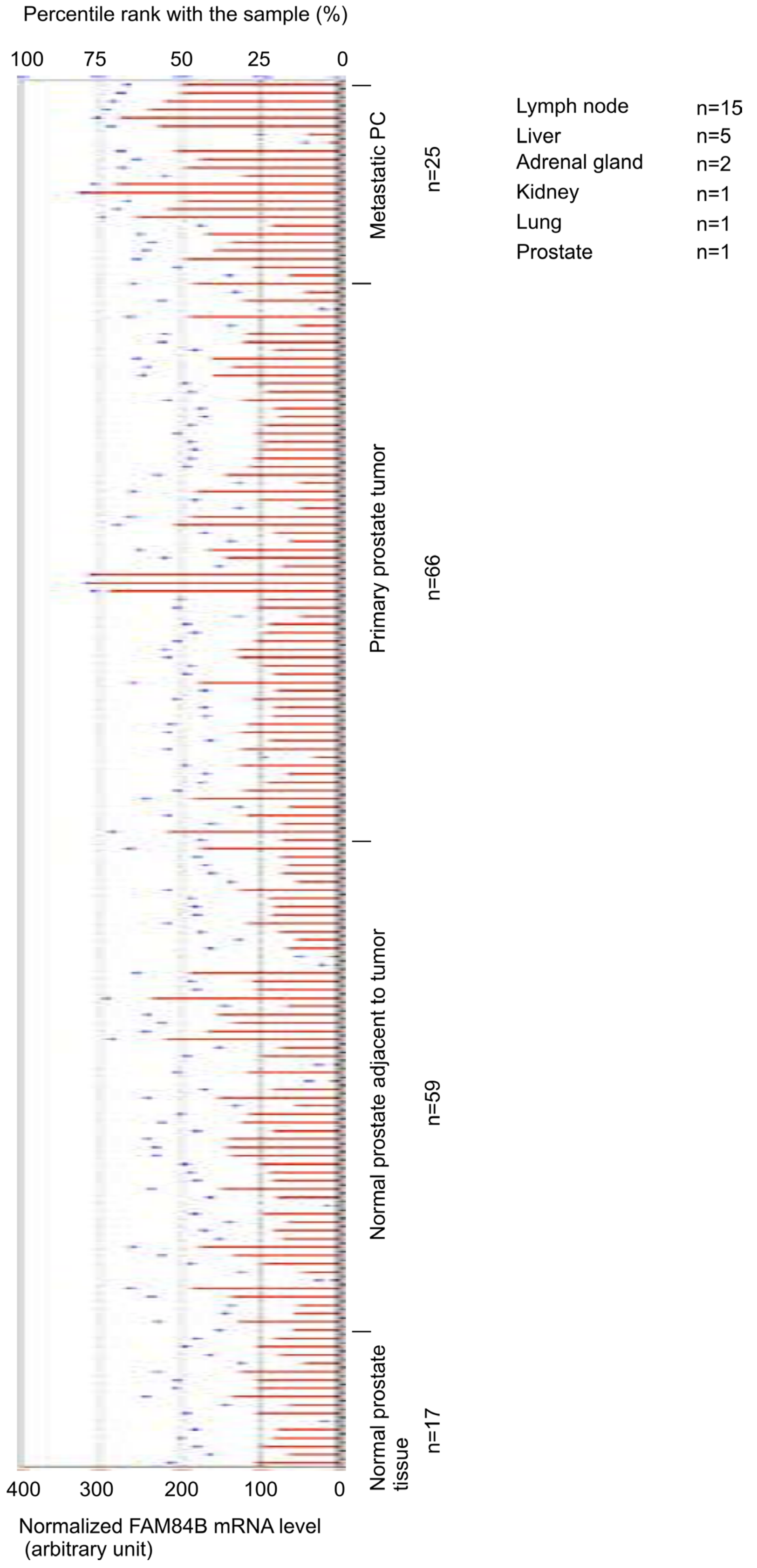

Supplementary Figure 4: FAM84B mRNA levels in normal prostate, primary PCs, and metastatic PCs. The illustration was extracted from the GDS2546 dataset (Gene Expression Omnibus). The composition of the 25 metastases is also included.

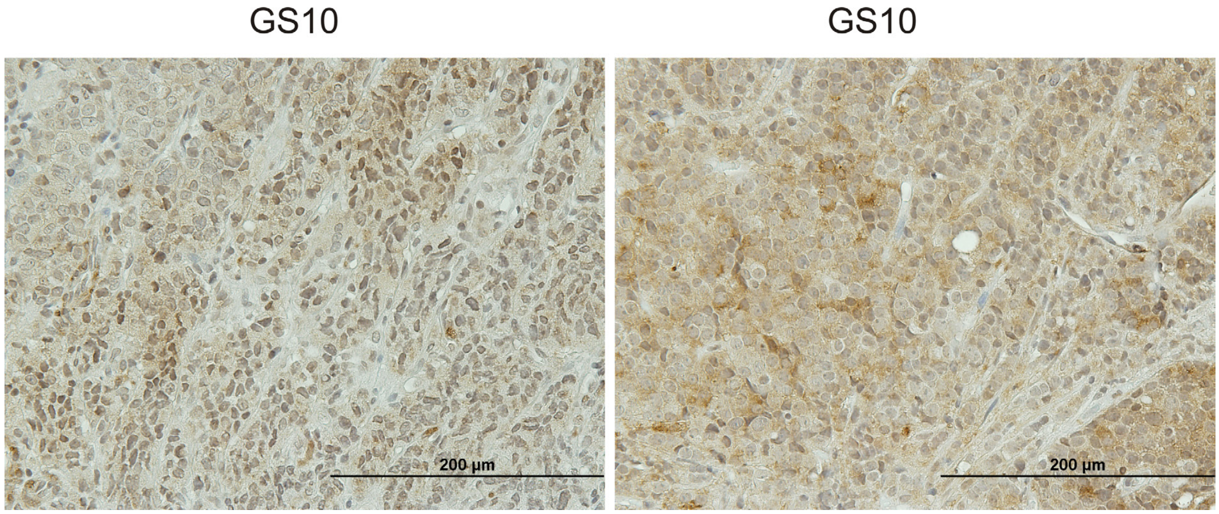

Supplementary Figure 5: FAM84B protein expression in primary prostate tumors. FAM84B staining in two Gleason score 10 prostate tumors.

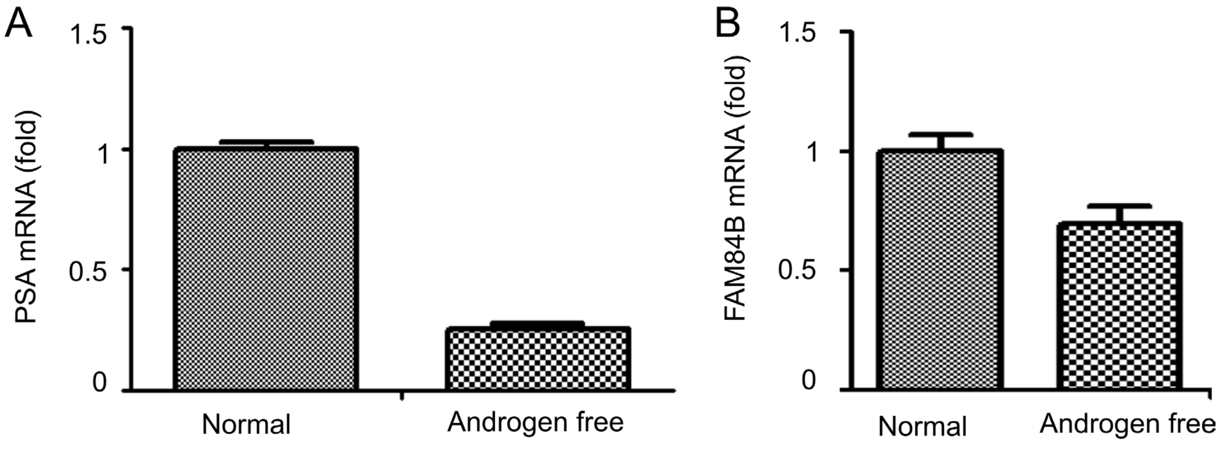

Supplementary Figure 6: Examination of FAM84B expression under androgen free conditions. LNCaP cells were cultured in normal or androgen free conditions for 24 hours, followed by real time PCR examination for PSA (**A**) and FAM84B mRNA (**B**). PCR reactions were carried out in triplicate; experiments were repeated once.

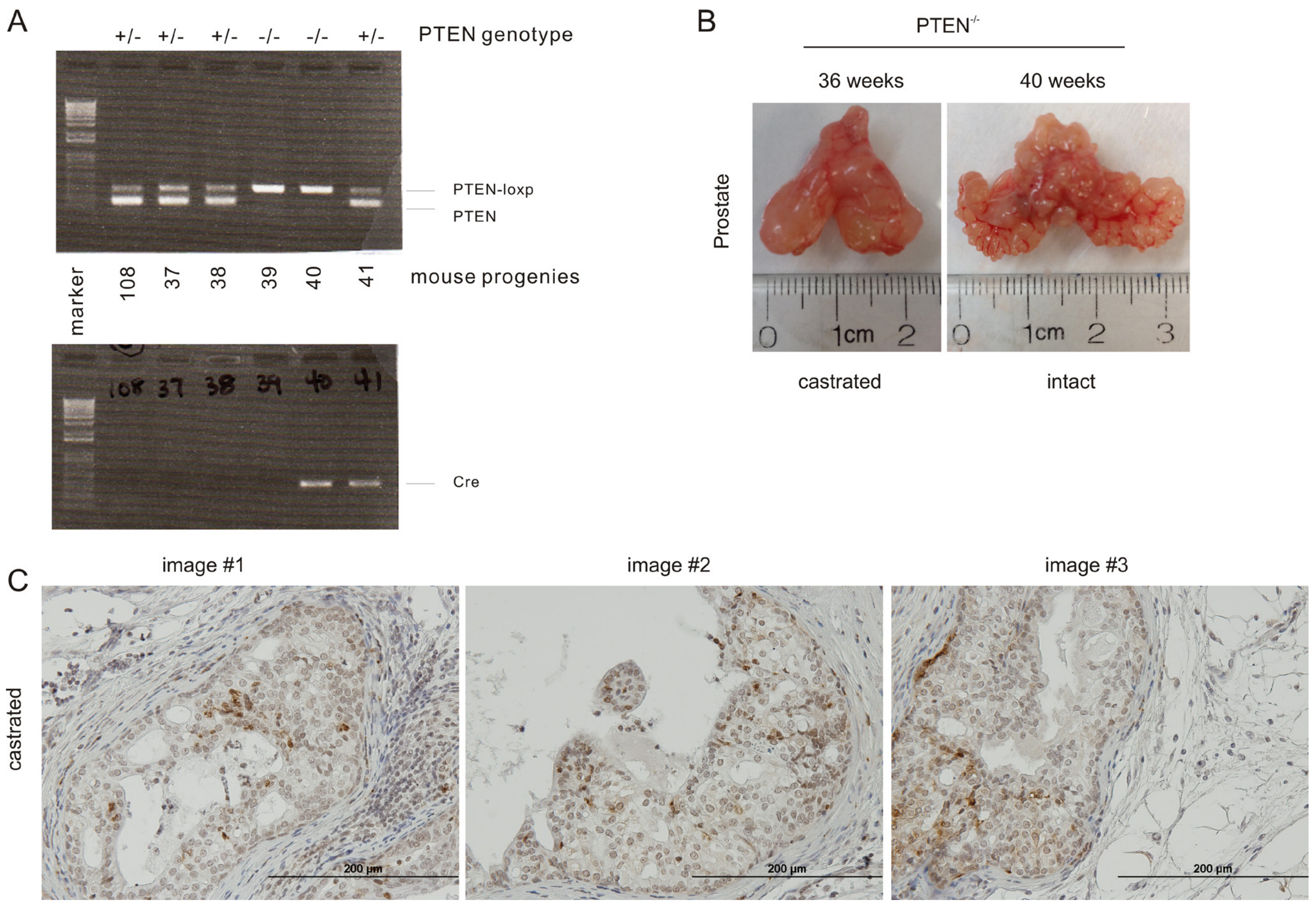

Supplementary Figure 7: Generation of castration resistance prostate cancer (CRPC) in prostate specific PTEN$^{-/-}$ mice. (**A**) Prostate specific PTEN$^{-/-}$ mice were produced by crossing PTEN$^{loxp/loxp}$ mice with PB-Cre4 (prostate specific expression of Cre) mice. Genotyping of six mouse progenies for the PTEN locus (top panel) and the Cre transgene locus (bottom panel) using tail genomic DNA. Pten$^{loxp/loxp}$ (Forward): 5'- CAAGCACTCTGCGAACTGAG -3', Pten$^{loxp/loxp}$ (Reverse): 5'- AAGTTTTTGAAGGCAAGATGC -3', PB-Cre4 (Forward): 5'- CTGAAGAAT GGGACAGGCATTG -3', PB-Cre4 (Reverse): 5'- CATCACTCGTTGCATCGACC -3'. (**B**) PTEN$^{-/-}$ mice were castrated at 23 weeks old for 13 weeks. Typical images of age-matched partial urogenital systems (bladder, prostatic lobes, seminal vesicles) from an intact and a castrated mouse are shown. (**C**) Different images from castrated mice.

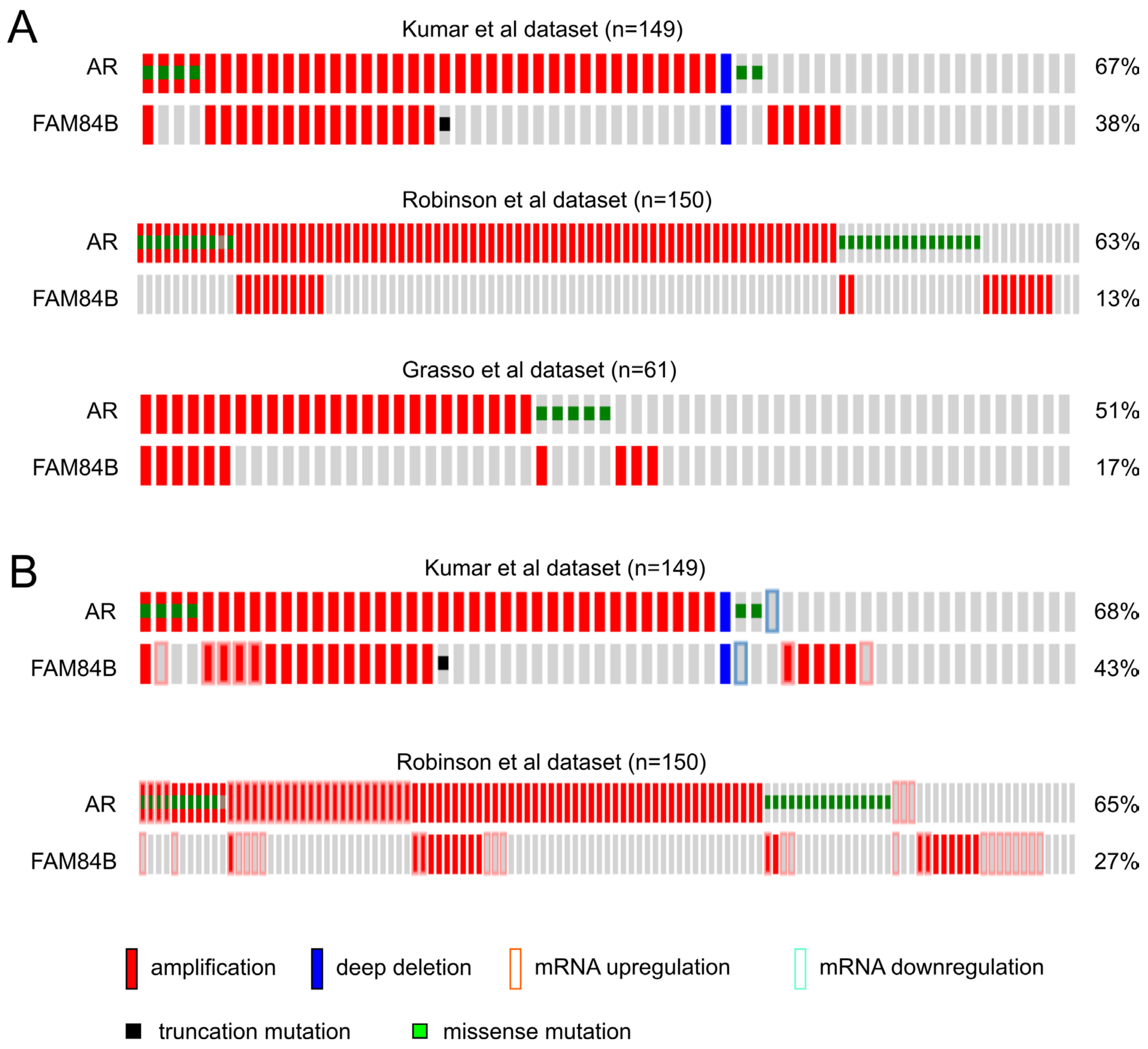

Supplementary Figure 8: Genomic alterations in the AR gene and FAM84B gene. Genomic changes alone (**A**) and with mRNA expression alterations (**B**) in the AR and FAM84B genes in the indicated datasets from the cBioPortal database.

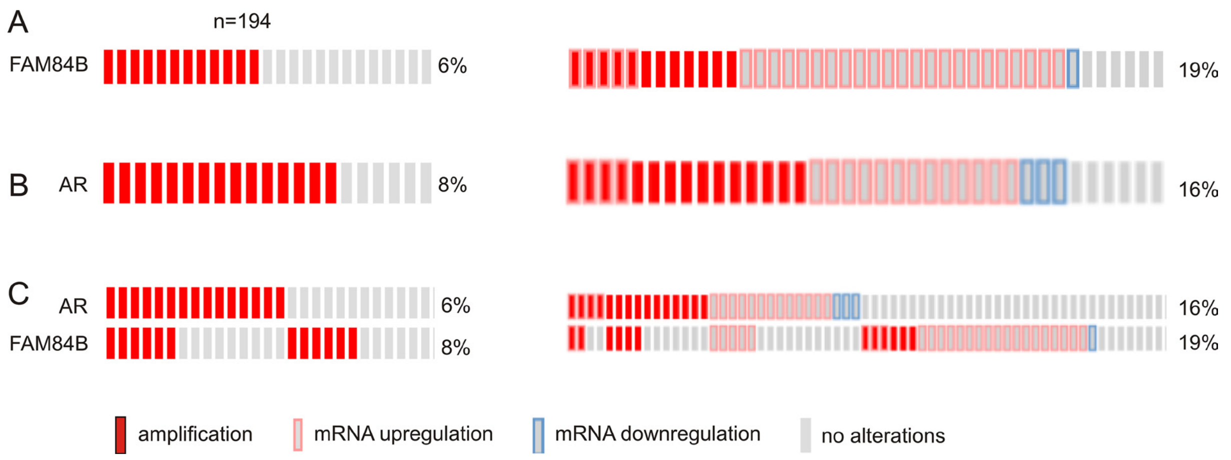

Supplementary Figure 9: Genomic alterations and mRNA expression changes in the AR and FAM84B genes. Data were extracted from the "MSKCC, Cancer Cell 2010" dataset within the cBioPortal database. Symbols for individual color bars are provided. “no alterations”: no change at either genomic level or mRNA expression.

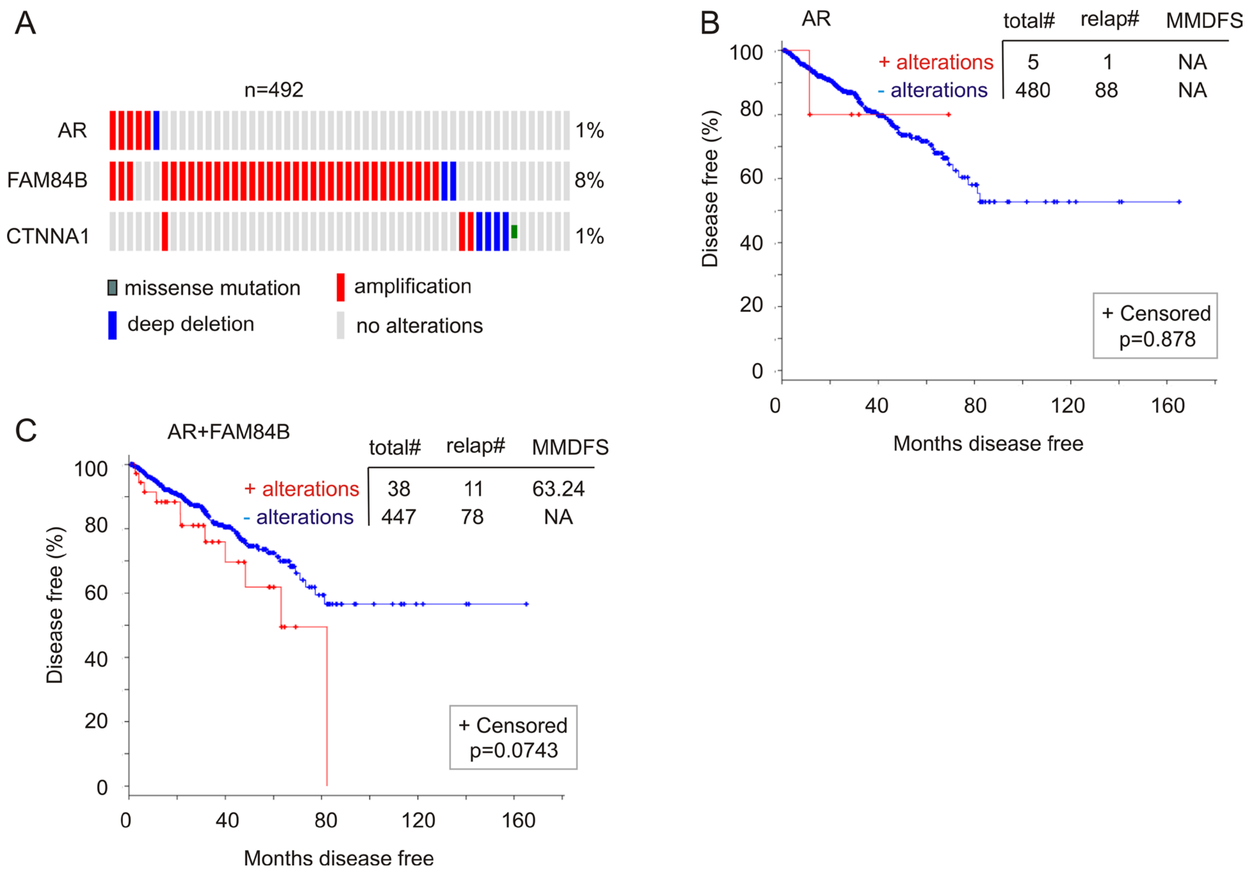

Supplementary Figure 10: The relationship of genomic changes in the AR, FAM84B, and CTNNA1 genes with disease free survival (DFS). Data for a 492 patient cohort with gene copy number variations determined were extracted from the TCGA dataset within the cBioPortal database. (**A**) Only the proportion of patients with the indicated genomic alterations is shown. Number of patients and the rates of genomic changes for the indicated genes are included. Symbols for individual color bars are provided. “no alterations”: no change at either genomic level or mRNA expression. (**B**, **C**) The association of AR or AR+FAM84B with DFS is shown. Statistical analysis was performed using Logrank Test.

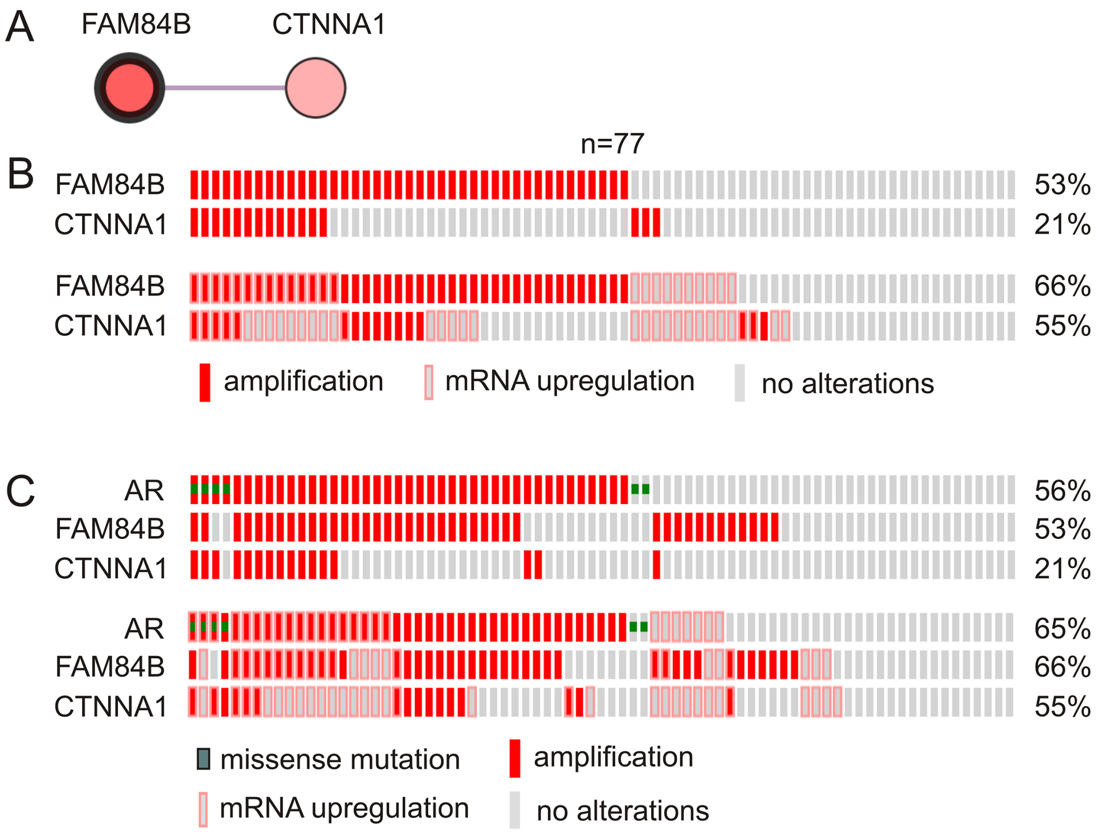

Supplementary Figure 11: The FAM84B network. (**A**) The network was generated using FAM84B as the seed node; the liner node was identified using the pathway data and interaction data from HPRD, Reactome, NCI-Nature Pathway Interaction Database, and the MSKCC Cancer Cell Map. The pathway was constructed by the system provided by cBioPortal (http://www.cbioportal.org/). (**B**, **C**) Gene amplifications with and without mRNA upregulation in the FAM84B network alone (B) and with AR (C) are shown. Data were extracted from the "Trento/Cornell/Broad 2016" dataset within the cBioPortal database. Symbols for individual color bars are provided. “no alterations”: no change at either genomic level or mRNA expression.
